# In silico comparative structural and functional analysis of arsenite methyltransferase from bacteria, fungi, fishes, birds, and mammals

**DOI:** 10.1186/s43141-023-00522-9

**Published:** 2023-05-19

**Authors:** Ashutosh Kabiraj, Anubhab Laha, Anindya Sundar Panja, Rajib Bandopadhyay

**Affiliations:** 1 Department of Botany, UGC-Centre for Advanced Study, The University of Burdwan, Golapbag, Bardhaman, West Bengal 713104 India; 2 Department of Botany, Chandernagore College, Hooghly, Chandernagore, West Bengal 712136 India; 3 Molecular Informatics Laboratory, Department of Biotechnology, Oriental Institute of Science and Technology, Vidyasagar University, Midnapore, West Bengal 721102 India

**Keywords:** Arsenite methyltransferase, *arsM*, *as3mt*, Homology modelling, SAM, SWISS-MODEL

## Abstract

**Background:**

Arsenic, a ubiquitous toxic metalloid, is a threat to the survival of all living organisms. Bioaccumulation of arsenic interferes with the normal physiological pathway. To overcome arsenic toxicity, organisms have developed arsenite methyltransferase enzyme, which methylates inorganic arsenite to organic arsenic MMA (III) in the presence of S-adenosylmethionine (SAM). Bacteria-derived *arsM* might be horizontally transported to different domains of life as *arsM* or *as3mt* (animal ortholog). A systematic study on the functional diversity of arsenite methyltransferase from various sources will be used in arsenic bioremediation.

**Results:**

Several arsenite methyltransferase protein sequences of bacteria, fungi, fishes, birds, and mammals were retrieved from the UniProt database. In silico physicochemical studies confirmed the acidic, hydrophilic, and thermostable nature of these enzymes. Interkingdom relationships were revealed by performing phylogenetic analysis. Homology modeling was performed by SWISS-MODEL, and that was validated through SAVES-v.6.0. QMEAN values ranged from − 0.93 to − 1.30, ERRAT score (83–96), PROCHECK (88–92%), and other parameters suggested models are statistically significant. MOTIF and PrankWeb discovered several functional motifs and active pockets within the proteins respectively. The STRING database showed protein–protein interaction networks.

**Conclusion:**

All of our in silico studies confirmed the fact that arsenite methyltransferase is a cytosolic stable enzyme with conserved sequences over a wide range of organisms. Thus, because of its stable and ubiquitous nature, arsenite methyltransferase could be employed in arsenic bioremediation.

**Supplementary Information:**

The online version contains supplementary material available at 10.1186/s43141-023-00522-9.

## Background

Arsenic, a ubiquitously found metalloid, has been ranked 20th, 14th, and 12th in the earth’s crust, seawater, and human body respectively based on its occurrence [1, 2]. Rock weathering, erosion, volcanic eruption, extensive mining, use of pesticides, etc. are notable causes of environmental arsenic contamination [1–5]. Different forms of arsenic exposure have led to drastic metabolic changes, even death, thereby affecting more than 300 million people in over 115 countries [3, 4]. Arsenic contaminants found in drinking water, food, or sometimes air cause skin, liver, lung, bladder cancers, and cardiovascular disease, mental disorder, etc. [5].

The two most common forms of arsenic, i.e., arsenite [As (III)] and arsenate [As (V)], enter through aquaglyceroporins and phosphate channels of bacteria respectively and interfere with their metabolism. These elements produce reactive oxygen species (ROS) in the cell, which leads to DNA damage or mutation and impairs enzymatic function [6, 7]. ATP synthesis gets disrupted due to abrupt changes in mitochondrial membrane potential in eukaryotes. Arsenic toxicity leads to the generation of nitric oxide (NO), superoxide ions (O_2_^−^), and hydroxyl radicals (OH), consequently triggering tumor formation [7].

Nowadays, arsenic-resistant bacteria and fungi are being deployed in bioremediation, due to their ability to perform biosorption, bioaccumulation, biotransformation [8, 9]. Though several living organisms ranging from bacteria to humans can methylate inorganic arsenic during detoxification, higher plants cannot do so [10]. Different methylated forms of arsenic, viz., monomethyl arsinic acids (MMAs), dimethyl arsinic acids (DMAs), trimethyl arsinic acids (TMAs), arsenosugars, arsenolipids, etc. are found in nature. Arsinothricin (AST) is a methylarsenical antibiotic that is used as a bacterial weapon to protect themselves from other strains. The presence of arsenic could be detected in the lipid extracts of fishes [11]. Yang et al. [12] have reported that food and water act as the mode of entry of arsenic in bird species. They detected the presence of arsenite, arsenate, DMA, MMA, etc. in the feathers and muscles of two birds inhabiting a highly arsenic-contaminated area of China [12]. In human beings, arsenite is at first methylated to MMA and is subsequently converted to DMA, the excreted form. Scientific studies have proved MMA to be more dangerous than DMA [1]. Even recent researches suggest methylated arsenic species to be a potent cause of women’s breast cancer [13].

*arsM* system has been developed in ancient bacterial strains to detoxify the harmful effects of arsenic. In the presence of SAM (S-adenosylmethionine), arsenite methyltransferase (encoded by *arsM* gene) can methylate arsenite. Horizontal gene transfer (HGT) plays a key decisive role in the development of resistance across various domains of living organisms [14]. Orthologs of *arsM* are observed in fungi and animals as *arsM* and *as3mt*, respectively. Arsenite methyltransferase contains three conserved domains, viz., N-terminal domain (that binds with SAM), middle domain (that deals with arsenite), and C-terminal domain (function unknown). Three to 4 conserved cysteine residues are very important for proper enzymatic function for all types of organisms. AS3MT enzyme is found in the liver of human beings [1].

Arsenic has been an infamous environmental pollutant from the beginning of time. Bioaccumulation and biomagnification of arsenic exhibit undesirable physiological changes in the living organisms, affecting their survival. Thus, bioremediation of this toxic metalloid is of utmost necessity in this predicament. The omnipresent arsenite methyltransferase will play a crucial role at this juncture. Comparative in silico analyses of this enzyme among different domains of life are not well studied to date. This present study will aid in understanding their inter-relationships and physiochemical characteristics (both structural and functional conservation of amino acids, etc.).

Arsenic methyltransferase from various sources was assessed for structural and functional properties to better understand the roles of arsenic bioremediation capability. This in silico study will thus help to develop a cost-effective and efficient method of utilizing arsenite methylarsenite as an arsenic bioremediation agent in future in vivo applications.

## Materials and methods

### Sequence retrieval

One-hundred fifteen amino acid sequences (i.e., 25 amino acid sequences of bacteria, 25 amino acid sequences of birds, 25 amino acid sequences of mammals, 20 amino acid sequences of fungi, and 20 amino acid sequences of fishes) of arsenite methyltransferase were retrieved in FASTA format from UniProtKB database (https://www.uniprot.org/) on 27th–30th May 2021.

### Primary sequence analysis and phylogenetic tree construction

ExPASy ProtParam (https://web.expasy.org/protparam/) was used to determine amino acid sequences, theoretical PI, aliphatic index, instability index, grand average of hydropathicity (GRAVY), etc. [15] of arsenite methyltransferase. Phylogenetic relationships among organisms were studied using MEGA-X software, and a phylogenetic tree was constructed based on 500 bootstrap values [16].

### Analysis of secondary structure

Hydrogen bonding between amino acids containing amide hydrogen and carbonyl oxygen is responsible for the construction of secondary structures in proteins. *α*-helix and *β*-sheets are common secondary structures within proteins. In this study, comparative analysis of secondary structures, viz., *α*-helix, *β*-turn, extended loop, and random coil, was done, after selecting five sequences for each organism by SOPMA (https://npsa-prabi.ibcp.fr/cgi-bin/npsa_automat.pl?page=/NPSA/npsa_sopma.html) web server tool [17].

### Analysis of tertiary structure

SWISS-MODEL workplace (https://swissmodel.expasy.org/) was used to predict the 3D structure of selected enzymes [18]. Total five structures were predicted (one structure per organism), and the most suitable templates were considered for modeling. Constructed models were then validated and reanalyzed by using another web server SAVES v6.0 (https://saves.mbi.ucla.edu/). The models were processed through the ERRAT server [19], PROCHECK [20], and VERIFY-3D [21] of SAVES v6.0 for qualitative analyses. Salt bridge combinations were detected by ESBRI (http://bioinformatica.isa.cnr.it/ESBRI/introduction.html) [22].

### Functional analysis of enzymes

PrankWeb server (https://prankweb.cz/) was used to predict probable ligand binding sites of the enzymes [23]. Sequence-based structural conservation was studied by MEGA [16]. Cofactory v.1.0 (http://www.cbs.dtu.dk/services/Cofactory/) server, a tool for identification of cofactor(s) related with enzymatic function, was implemented [24]. Protein localization within the cells was confirmed by SignalP 5.0 (http://www.cbs.dtu.dk/services/SignalP/) server [25]. Transmembrane helices were predicted using TMHMM server v. 2.0 (http://www.cbs.dtu.dk/services/TMHMM/) [26]. Related motifs were searched of these enzymes through MOTIF tool (https://www.genome.jp/tools/motif). Finally, protein–protein interaction networks were studied by STRING database (https://string-db.org/) [27].

## Result

### Sequence retrieval

Amino acid sequences of arsenite methyltransferase enzymes of 25 bacteria, 20 fungi, 20 fishes, 25 birds, and 25 mammals were retrieved from UniProtKB, and their protein accession numbers with respective organisms are documented in Supplementary data 1.

### Primary sequence analysis and phylogenetic tree construction

**Fig. 1:**
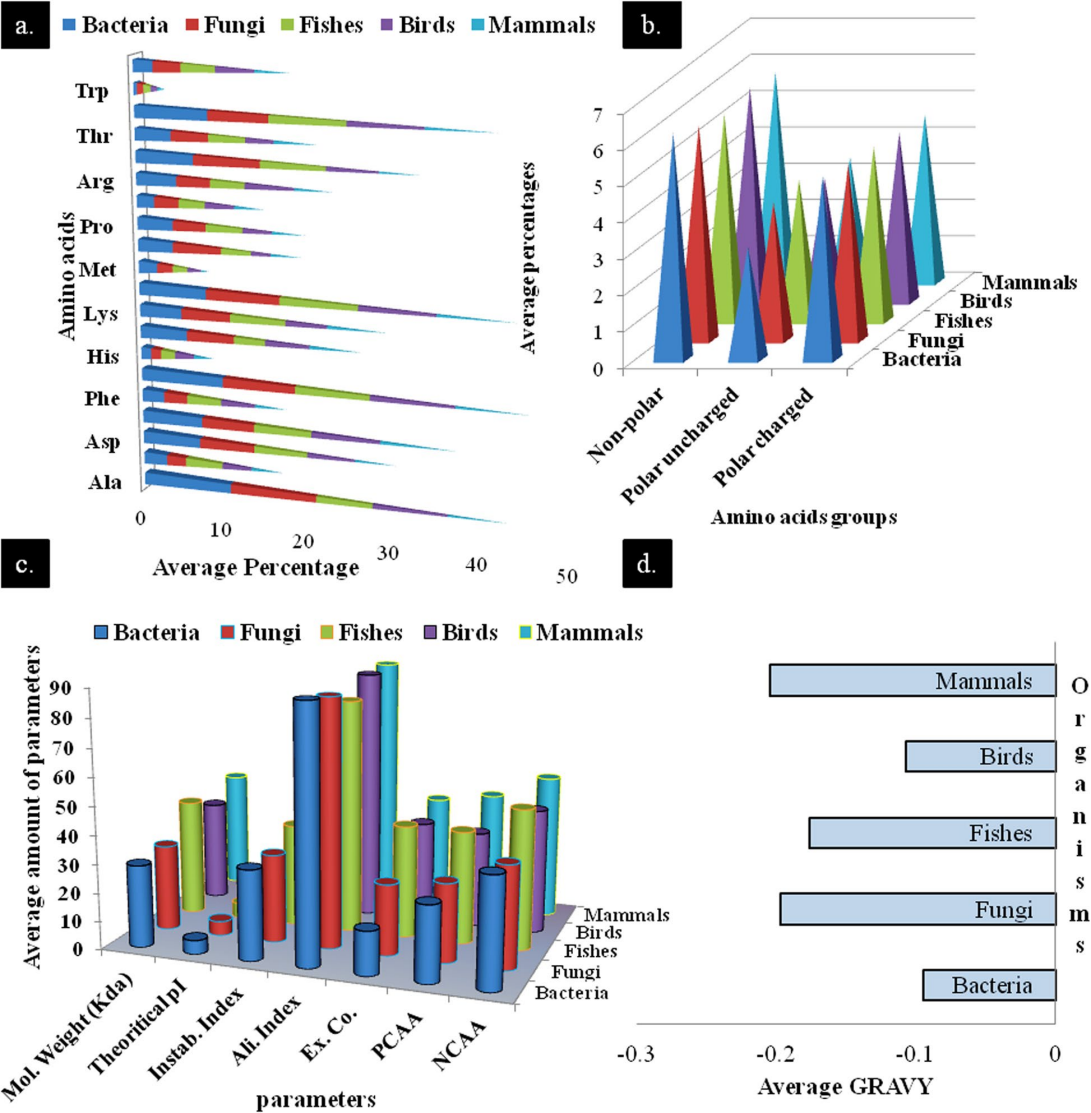
Amino acids composition and physicochemical parameters analysis of protein sequences of different organisms. **a** Average percentages of each amino acid. **b** Average percentages of nonpolar uncharged, polar uncharged, and charged amino acids. **c** Physicochemical parameters analysis (Instab. Index, instability index; Ali. Index, aliphatic index; Ex. Co., extinction coefficient (× 1000); PCAA, positively charged amino acids; NCAA, negatively charged amino acids). **d** Average GRAVY analysis

Amino acids are considered as building blocks for any protein. Thus, determination of the number and position (i.e., sequence) of amino acids is crucial factors for any structural and functional properties of a protein. Among 20 amino acids, the highest average concentration was found for alanine in arsenite methyltransferase. Bacteria contained the highest percentage of alanine (> 10%), and mammals showed minimum value (< 7%). The lowest concentration of amino acid goes to tryptophan (< 1%) for all the organisms (Fig. 1a). The average percentages of nonpolar uncharged amino acids were higher than the rest two, whereas polar charged and polar uncharged amino acid concentrations are moderate and least respectively.

However, the total average percentage of polar amino acid concentration (7–8%) is quite higher than nonpolar residues (6–7%), indicating that protein is slightly hydrophilic in nature (Fig. 1b). Average molecular weight of the proteins varies from about 29 to 42 kDa (Fig. 1c) which is quite less or equal to another arsenic detoxification enzyme, arsenite oxidase [28]. Average pI of proteins varies from 4.8 to 5.7 which indicates arsenite methyltransferase is slightly acidic in nature. The highest and lowest pI values were observed in case of a bird species, *Rhodinocichla rosea* (6.5), and bacteria, *Mumia* sp. (4.33) (Supplementary data 2) respectively. Among 115 protein sequences, only 6 have instability index (II) more than 40. Report suggested that II value lower than 40 indicates stable proteins. Aliphatic index is the volume occupied by aliphatic side chains of a protein of aliphatic amino acids: alanine, valine, isoleucine, and leucine. Average aliphatic index of the proteins is above 80% confirming their thermostability [15, 29]. Negatively charged amino acids (NCAA) are more in number than positively charged amino acids (PCAA). A negative GRAVY value (Fig. 1d) indicates the hydrophilic nature of the protein [28].

**Fig. 2:**
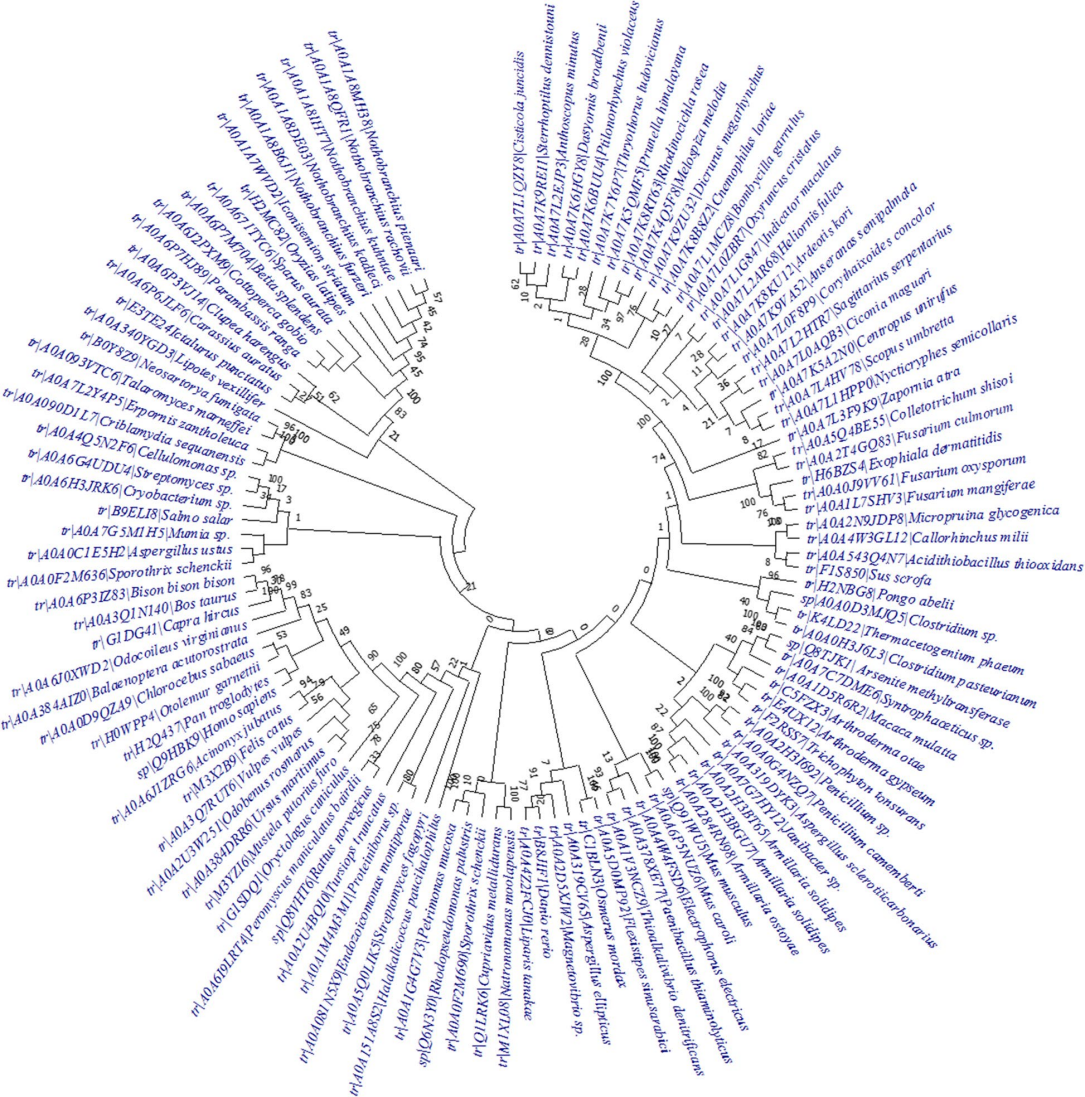
Phylogenetic tree of protein sequences of different organisms

Enzyme of *Mus* sp. is showing a separate lineage in the phylogenetic tree (Fig. 2). The arsenite methyltransferase enzymes of *Zapornia atra*, a bird, also separated itself from other bird lineages. Interestingly, arsenite methyltransferase enzymes of *Arthroderma otae* and *Arthroderma gypseum* are belonging to the same clade, while *A*. *gypseum* shared the same ancestor with *Trichophyton tonsurans*, although both the genera belong to Arthrodermataceae. Close inter-relationships could be noticed between the proteins of *Homo sapiens* and *Pan troglodytes*. This complex relationship indicates complex evolutionary relationships among different organisms. Phylogenetic trees for individual organisms were constructed which are represented in Supplementary data 3.

### Analysis of secondary structures

**Fig. 3:**
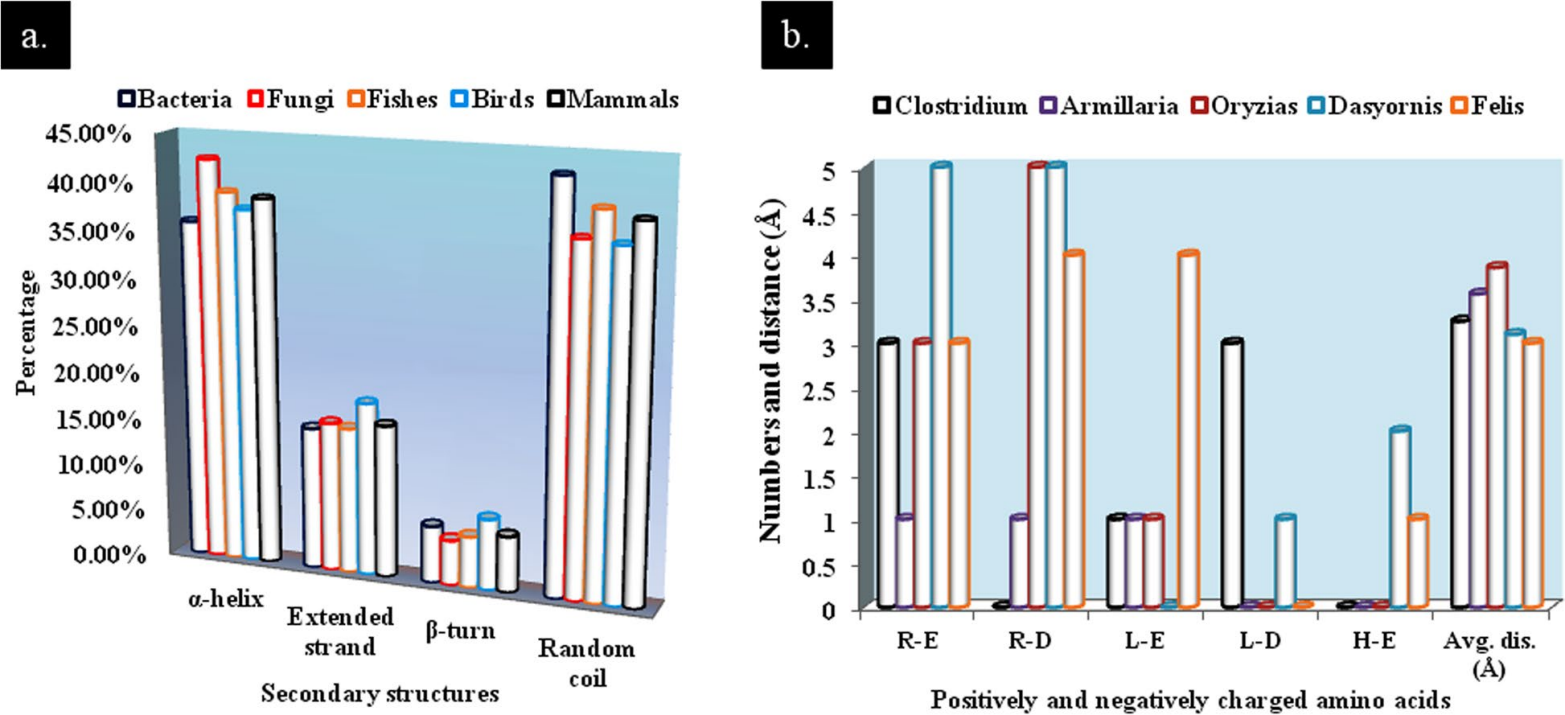
**a** Predictions of secondary structures of enzymes and **b** numbers and average distances of salt bridges of selected enzymes. Average distances are given in Å (R, arginine; E, glutamic acid; D, aspartic acid; L, lysine; H, histidine)

The average comparative secondary structures such as *α*-helix, *β*-turn, extended loop, and random coil were computed and graphically represented in Fig. 3a. *α*-helix and random coils were found to be most abundant. For fungi, average percentage of occurrence of *α*-helix is more than 42%, while in mammals it is the lowest, 36%. Among three helical structures (*α*-helix, *β*-turn, and 3_10_-helix), *α*-helix is most stable structure. Proline is an *α*-helix breaker, and the presence of excess amount of proline may lead to aperiodic protein structure [30]. Excessive random coils indicate that protein showed many conserved regions with evolutionary significance. Random coils sometimes give more flexibility to protein [31].

### Analysis of tertiary structure

**Fig. 4:**
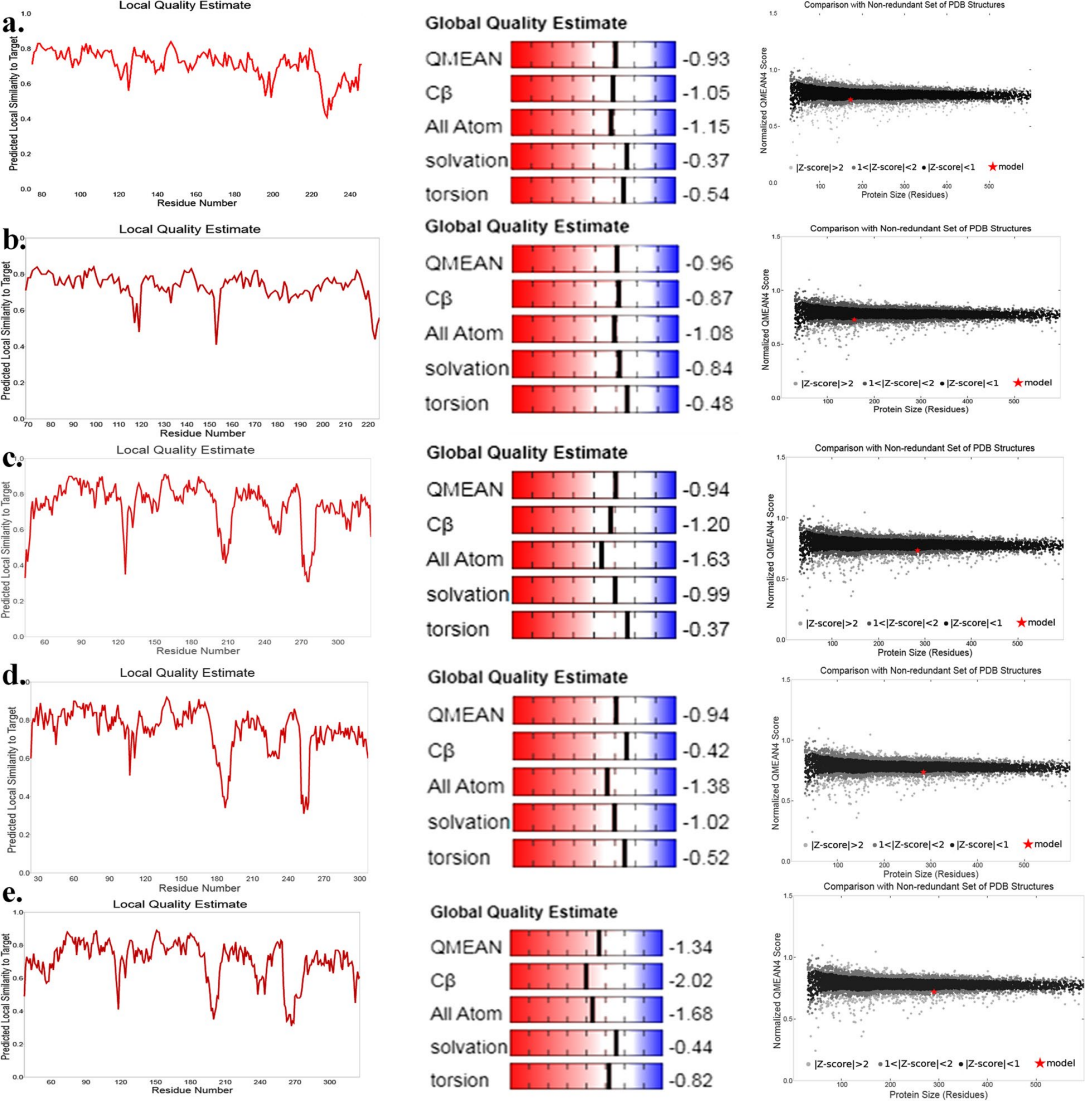
Local, global quality, and Z-score estimations of different organisms. **a ***Clostridium*, **b ***Armillaria solidipes*, **c ***Oryzias latipes*, **d ***Dasyornis broadbenti*, and **e ***Felis catus*

All sequences of arsenite methyltransferase enzymes were analyzed through SWISS-MODEL workplace for selecting the best suitable sequences on basis of QMEAN score: bacteria (QMEAN − 4.33 to − 0.93); fungi (QMEAN − 5.54 to − 0.46); fishes (QMEAN − 3.68 to − 0.94); birds (QMEAN − 3.29 to − 0.94); and mammals (QMEAN − 1.34 to 2.90). Among them, the arsenite methyltransferase enzymes of bacterium *Clostridium* sp. (− 0.93); fungus *Armillaria solidipes* (− 0.96); fish *Oryzias latipes* (− 0.94); bird *Dasyornis broadbenti* (− 0.94); and mammal *Felis catus* (− 1.34) were taken for further analysis (Fig. 4). ERRAT quality factors ranged from 83 to 96 (Table 1) confirming the high resolutions of the protein structures. Here, in this experiment, proteins of bacteria and fungi displayed acceptable results (Supplementary data 4 and Table 1).

**Table 1:** Quality estimations of proteins of different organisms

**Organisms**	**QMEAN scores**	**ERRAT values**	**Ramachandran plot**^a^	**VERIFY-3D scores**
*Clostridium* sp.	− 0.93	95.455	89.9%	93.60%
*Armillaria solidipes*	− 0.96	96.528	92.4%	82.80%
*Oryzias latipes*	− 0.94	85.609	87.3%	98.24%
*Dasyornis broadbenti*	− 0.94	83.212	90.3%	100%
*Felis catus*	− 1.34	90.357	88.2%	96.55%

^a^ Results are given on the basis of most favored regions

Among five organisms, VERIFY-3D revealed the least average 3D-1D score gained in fungi (82.80%), whereas others showed good scores (Table 1). A total of 90% or more residues occurred in the most favored regions in the Ramachandran plot, thereby indicating good protein model quality of *Armillaria* and *Dasyornis* (Table 1). However, other models showed more than 95% residues in allowed region too (Supplementary data 5). Additionally, Ramachandran plots of proteins, Chi1-Chi2 plots, and Ramachandran plots of individual amino acids have been provided in Supplementary data 6 and 7. Salt bridges are the interactions between side chains of a protein-associated positively (Lys, His, and Arg) and negatively charged (Asp and Glu) amino acids between the bond distances of 7 Å. There are several salt bridges present as suggested by ESBRI server [22] (Fig. 3b). The protein sequence of a bird, *Dasyornis* sp., had maximum pair of charged amino acids involved in salt bridges formation (i.e., 13 amino acids), while fungus *Armillaria* sp. had a minimum number of amino acids involved (i.e., 3 amino acids). Most dominating salt bridges are Arg-Asp, whereas only His-Asp salt bridge is present in *F*. *catus*.

### Functional analysis of the enzyme

**Fig. 5:**
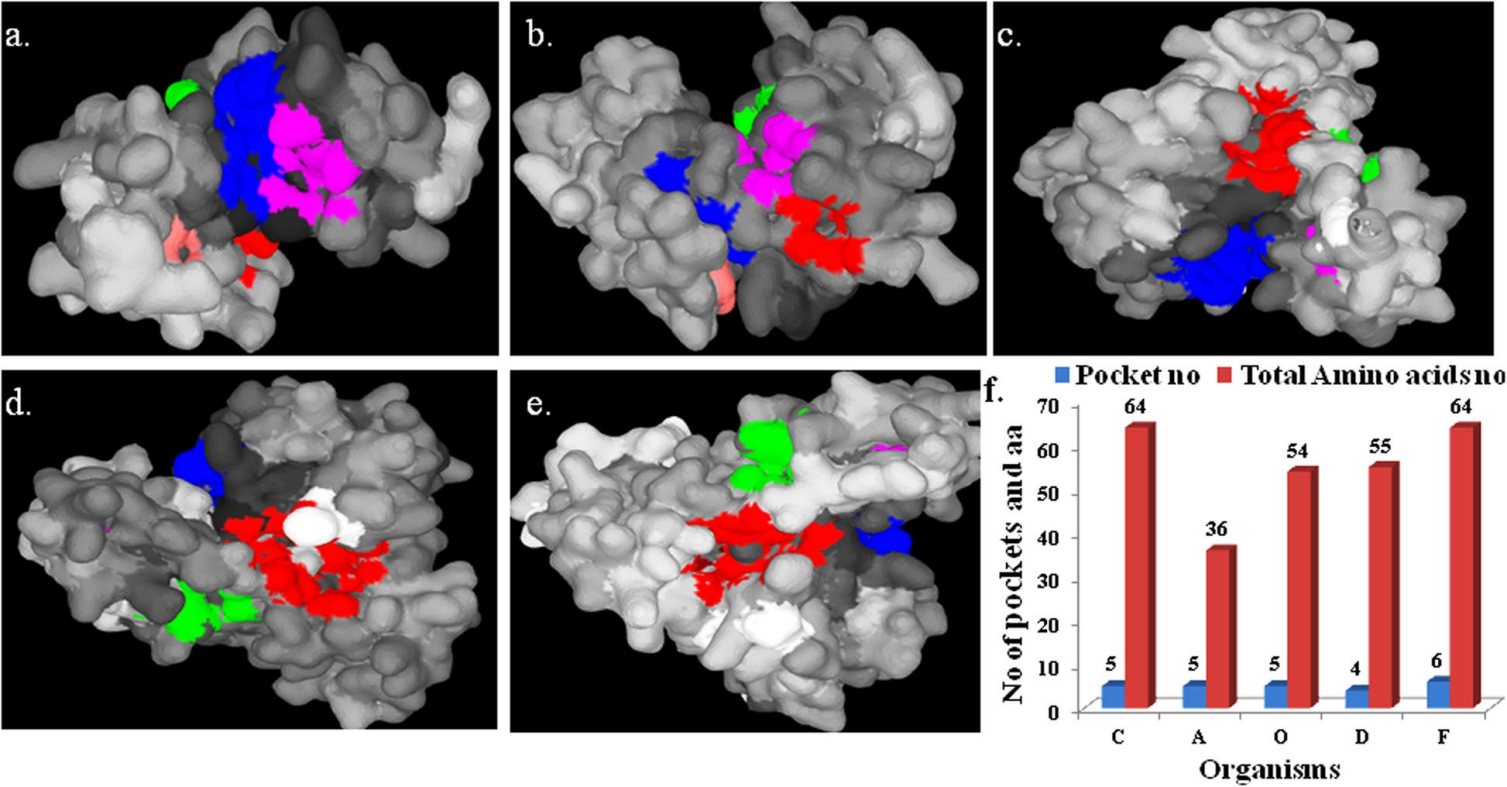
PrankWeb results showing active pockets (colored regions) of the enzymes of **a ***Clostridium* sp., **b ***Armillaria solidipes*, **c ***Oryzias latipes*, **d ***Dasyornis broadbenti*, **e ***Felis catus*, and **f** total number of pockets and involved amino acids of different organisms (C, *Clostridium*; A, *Armillaria*; O, *Oryzias*; D, *Dasyornis*; and F, *Felis*.)

PrankWeb results showed that within the enzyme, though several numbers (4–6) of functional pockets are present, the numbers of amino acids involved in pocket formation remain approximately the same (except in the case of *Armillaria* sp., where 36 amino acids lead to the formation of 5 pockets) (Fig. 5f). Pockets are dominated by charged or polar (serine, threonine, cysteine, etc.), occasionally nonpolar (isoleucine, leucine, proline, or glycine) amino acids. Cysteine, which has a pivotal role in arsenite methylation, was found either in pocket 1 or 2 or in both pockets in case of all enzymes, except that of *Armillaria* sp. (Fig. 5a–e). Local alignment of five sequences using the MEGA software revealed that there exists much significant sequence similarity among the amino acid sequences of the enzymes. By using MUSCLE algorithm of MEGA and based on 100% conservation sites, many conserved amino acids within the amino acid position of 94–103 were revealed (Supplementary data 8). On the other hand, only a few conserved sequences could be found within positions 167–210 in a scattered manner. Occasional swapping of positively charged amino acids could be observed too. For example, the 200th amino acid of the MUSCLE algorithm alignment, arginine (R) replaced lysine (K) in case of fungi, *Armillaria* (Supplementary data 8). Interestingly, high sequence conservation was observed among bacteria and fungi, signifying their close evolutionary lineage. Cofactors are chemical compounds or metal ions that are associated with enzymes for proper functioning. Cofactory v.1.0 web server can find out FAD, NAD, or NADH cofactors (if) present in enzyme [24]. However, no cofactors were found related to the enzyme. After translation, a signal peptide present in the N-termini of protein guides the protein to its target location. SignalP 5.0 finds out signal peptide along with its position in the protein, but here, no such signal peptide was discovered (Supplementary data 9). Additionally, TMHMM server did not find any transmembrane domain in it (Supplementary data 10) which also supports its subcellular localization. On the basis of these results, we can consider arsenite methyltransferase to be a cytosolic enzyme. MOTIF search (Supplementary data 11) revealed the existence of several functional domains of which the methyltransferase domain was the most predominant one. This domain of protein interacts with SAM and arsenite, where SAM donates methyl groups to arsenite; as a result, methylated arsenic species are generated [32]. STRING analysis reveals probable overall interactomics of the enzymes with different proteins, enzymes, etc. Animal arsenite methyltransferases (AS3MT) and their interactions with other proteins are well documented in Supplementary data 12.

## Discussion

Arsenic became one of the most threatening hazards since early origin of life [about 4 billion years ago (bya)] on earth, and its concentration drastically increased in the late Archean eon (3–2.5 bya). To fight against arsenic toxicity, early organisms developed ArsM enzyme which promoted defense mechanism for respective microorganisms. Gradually, after the Great Oxidation Event (GOE, 2.45–2.32 bya), it evolved as an arsenic-detoxifying enzyme [14]. Chen et al. [14] also argued that at least six horizontal gene transfers of *arsM* gene occurred between different kingdoms of life, resulting in a high diversity of inorganic arsenic-methylating species. Methylation and volatilization are enormously important and satisfactory mechanism adopted by bacteria and fungi for arsenic bioremediation [33–35]. Here, the comparison of arsenite methyltransferase from five groups of organisms, i.e., bacteria, fungi, fishes, birds, and mammals, was performed. A fungal species *Armillaria solidipes* showed close similarity with the bacteria *Janibacter* sp. (Fig. 2), thereby indicating the probability of horizontal gene transfer between two different kingdoms. Thermostable proteins are very important for industrial as well as in situ bioremediation applications. High aliphatic index (AI) (72–97) confirmed the thermostable nature of the proteins [29]. *α*-helix, the key regulator of protein stability, is found to occur more frequently in the proteins of thermophilic organisms than those of mesophilic ones [36]. In this study, *α*-helix occupied more than 35% (average) of the protein structure leading to good thermostability which is quite higher than extended strand and *β*-turn (Fig. 3a). Other physicochemical parameters, such as pI and instability indexes (Supplementary data 2), are important features for laboratory-based protein isolation and purifications. For homology modeling, in SWISS-MODEL workplace, most suitable models (confirmed by SAVES 6.0 server) were selected for subsequent alignment [viz., PDB ID: 4FR0 (for *Clostridium*); PDB ID: 4KW7 (for *Armillaria*) and PDB ID: 5EVJ (for three animals)] (Supplementary data 13). Former two are the proteins of a red alga, *Cyanidioschyzon*, whereas 5EVJ is identified as CrArsM of *Chlamydomonas reinhardtii*. Addition or deletion of salt bridges within protein structure may decrease or increase its stability respectively. Carboxyl oxygen of negatively charged amino acids interacts with nitrogen atoms of positively charged amino acids when they are present within a 4.0 Å distance either in the same polypeptide sequence or different [22]. In the present study, lysine and arginine are common positively charged amino acids involved in salt bridge formation. STRING analysis revealed that every enzyme has significantly more interaction probabilities than expected and showed first and second shells of interactions (Supplementary data 12). Bacterial ArsM interacts with arsenate reductase (ArsC), another arsenic-detoxifying enzyme responsible for reduction of arsenate to arsenite [37], and also some permeases which pump out arsenicals from cells. The fungal protein interacts with calmodulin, chaperones, ArsH, involved in enzymatic transformations of methylated arsenite [14], etc. Among animals, fish protein (AS3MT) interacts with aquaglyceroporins, sometimes related with arsenite import into the cell. It was also found to be interconnected with mitochondrial energy-producing enzymes by both first and second shells of interactions. Bird AS3MT shows similarities with fish AS3MT; additively, it interacts with some defense-related proteins. Finally, *Felis catus* enzyme showed active participation in glutathione metabolism. For animals, every AS3MT interacts with mitochondria-associated proteins involved in energy generation. So, there would be a relationship between arsenite biotransformation and energy generation within cells. For animals interactome, one enzyme, putative N-6 adenine-specific DNA methyltransferase 1 (N6AMT1), is common, which transforms monomethylarsonous acid to dimethylarsinic acid [38]. Besides humans, this enzyme is also present in fishes and birds confirming the evolutionary conservation of the enzyme.

## Conclusion

Arsenite methyltransferase is a thermostable, hydrophilic, evolutionary conserved enzyme involved in arsenite biotransformation (i.e., methylation). This in silico study sheds some light into the probable role of the amino acids in arsenite methylation. The conserved sequences and domains among different classes of arsenite methyltransferase promote its employability in bioremediation over different ecologies.

## Supplementary Information

**Additional file 1.** List of organisms with protein accession numbers.

**Additional file 2.** Physiobiochemical analysis of enzymes: ExPASy.

**Additional file 3.** Phylogenetic trees of different organisms.

**Additional file 4.** ERRAT values of proteins.

**Additional file 5.** Ramachandran plots of individual organisms.

**Additional file 6. **Chi1-Chi2 scores of selected enzymes regarding individual amino acids.

**Additional file 7.** Ramachandran plots of individual amino acids of selected enzymes.

**Additional file 8. **Structural conservation among the sequences.

**Additional file 9.** Identification of probable signal sequences within enzymes.

**Additional file 10.** Studies on probable transmembrane helixes within enzymes.

**Additional file 11.** Identification of probable motifs.

**Additional file 12.** Results of STRING analysis.

**Additional file 13.** Alignment of most suitable enzymes with the query sequence.

## Data Availability

All data generated or analyzed during this study are included in this published article [and its supplementary information files].

## Abbreviations

Ali. Index: Aliphatic index
DMA: Dimethylarsinic acid
ESBRI: Entrepreneurship and Small Business Research Institute
Ex. Co.: Extinction coefficient
FAD: Flavin adenine dinucleotide
GRAVY: Grand average of hydrophobicity
HGT: Horizontal gene transfer
II: Instability index
MEGA: Molecular Evolutionary Genetics Analysis
MMA: Monomethyl arsinic acid
NAD: Nicotinamide adenine dinucleotide
NADH: Reduced nicotinamide adenine dinucleotide
NCAA: Negatively charged amino acids
PCAA: Positively charged amino acids
PDB: Protein Data Bank
pI: Isoelectric point
QMEAN: Qualitative Model Energy ANalysis
ROS: Reactive oxygen species
SAM: S-Adenosylmethionine
TMA: Trimethyl arsinic acid
