# Supplementary material for: In silico comparative structural and functional analysis of arsenite methyltransferase from bacteria, fungi, fishes, birds, and mammals
Source: J Genet Eng Biotechnol. 2023 May 19;21:64. doi: 10.1186/s43141-023-00522-9 (PMC10199152; doi:10.1186/s43141-023-00522-9)

## Slide 1
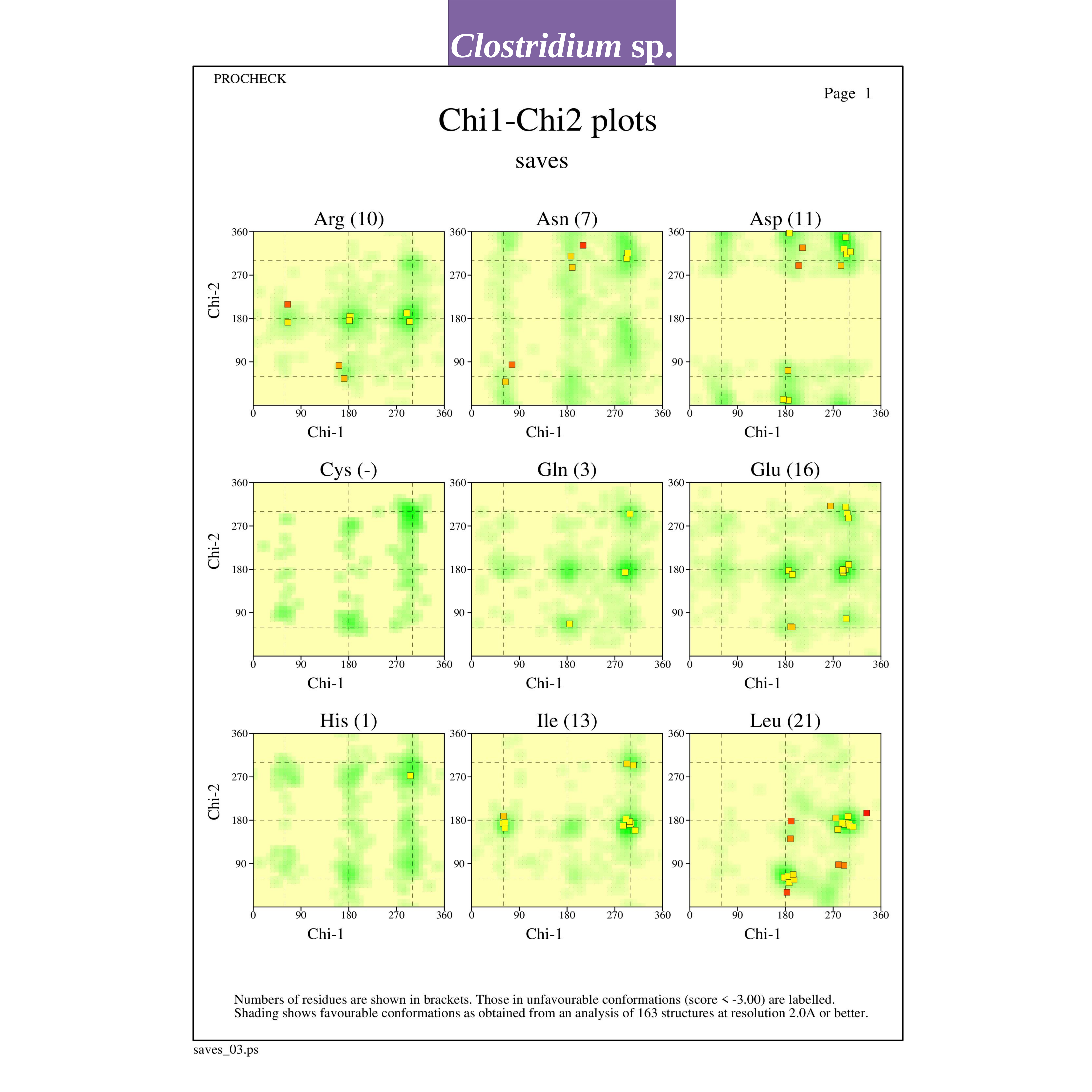

Clostridium sp.

## Slide 2
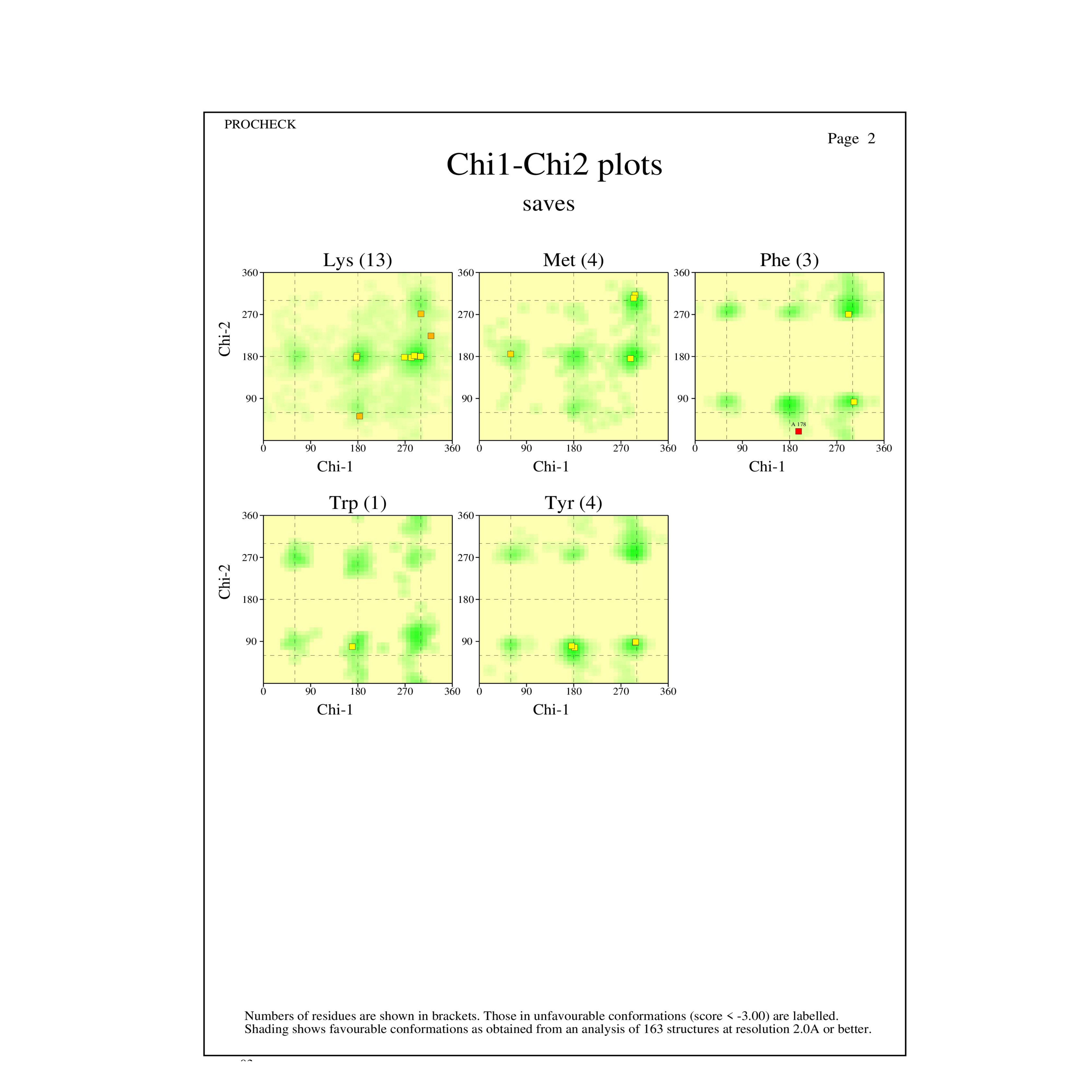

## Slide 3
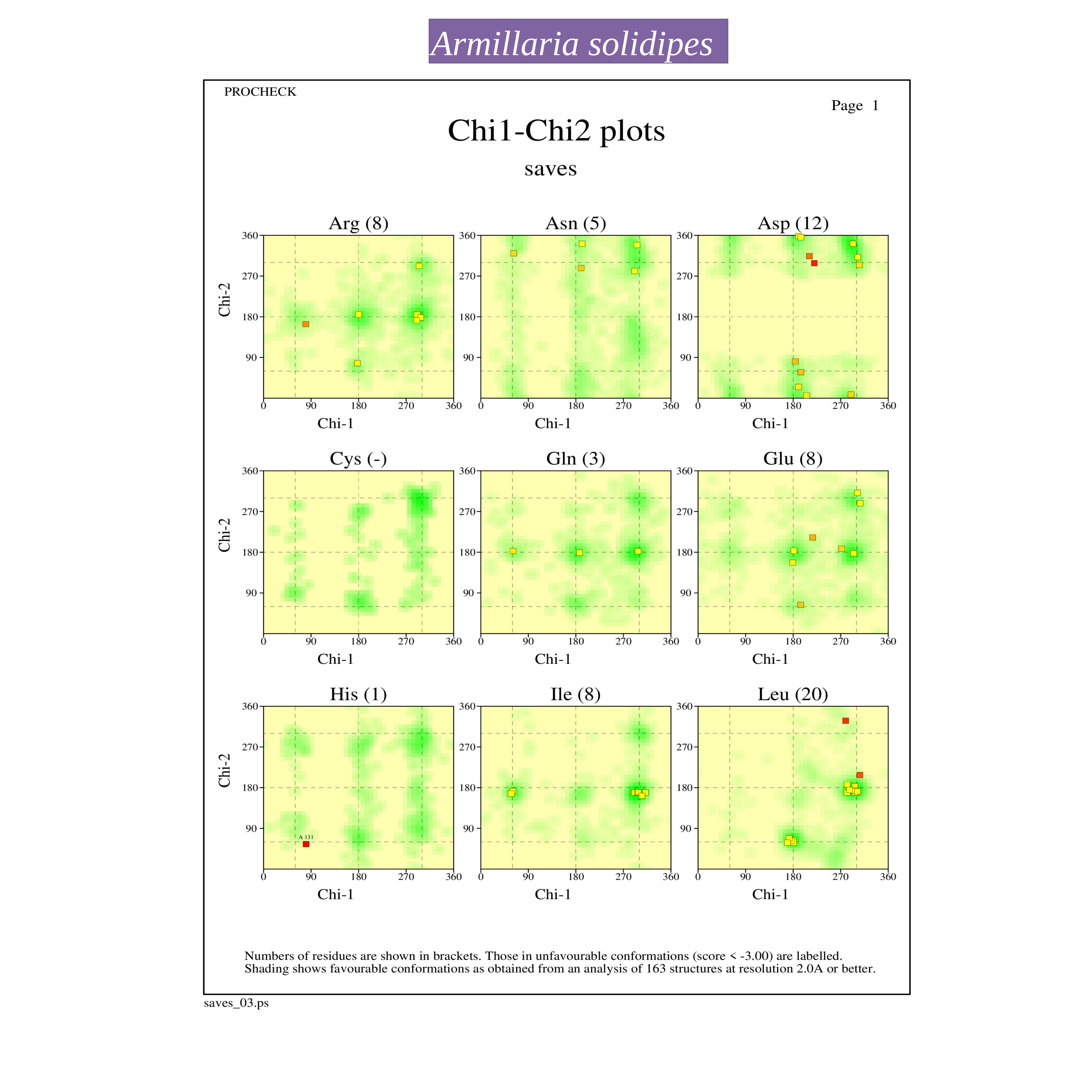

Armillaria solidipes

## Slide 4
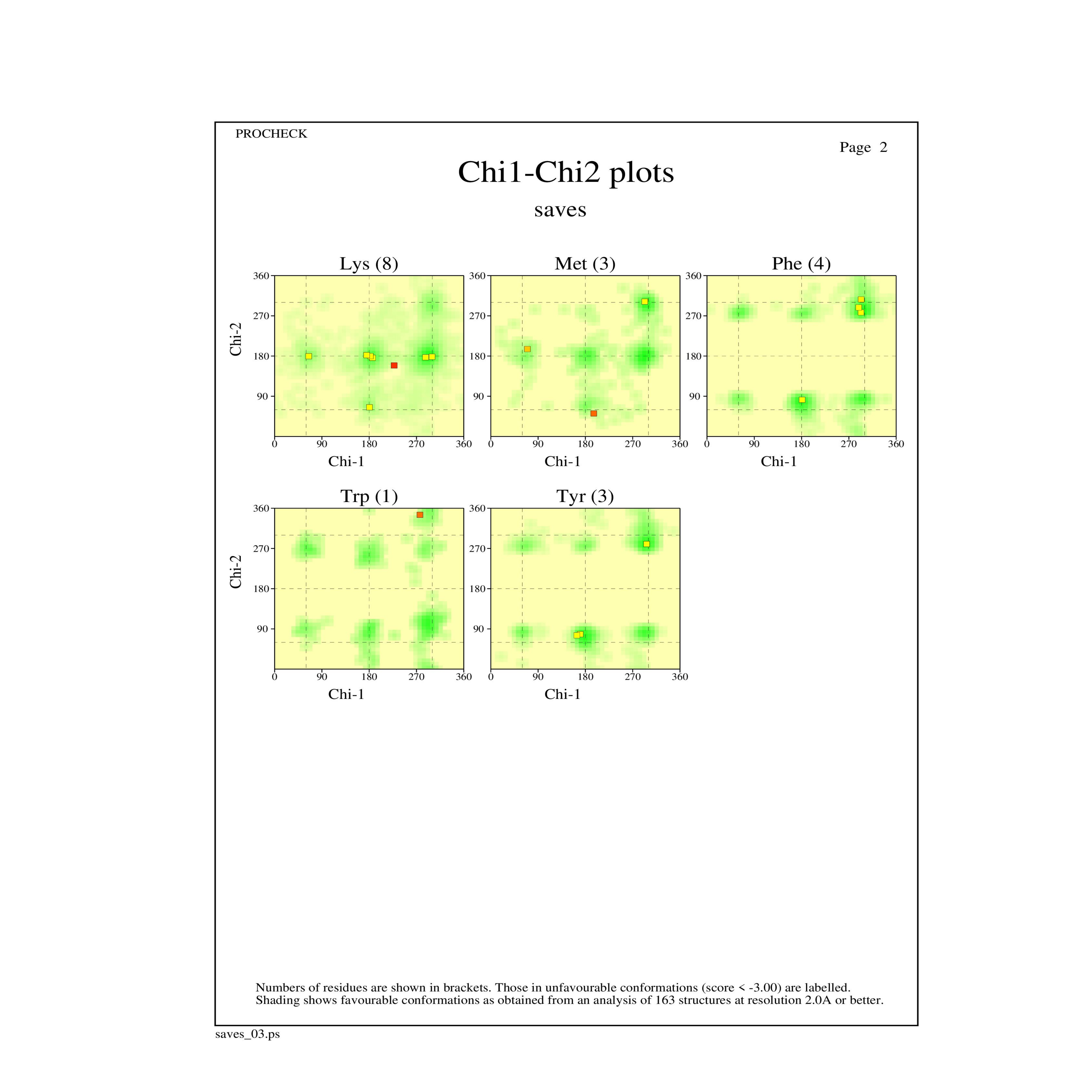

## Slide 5
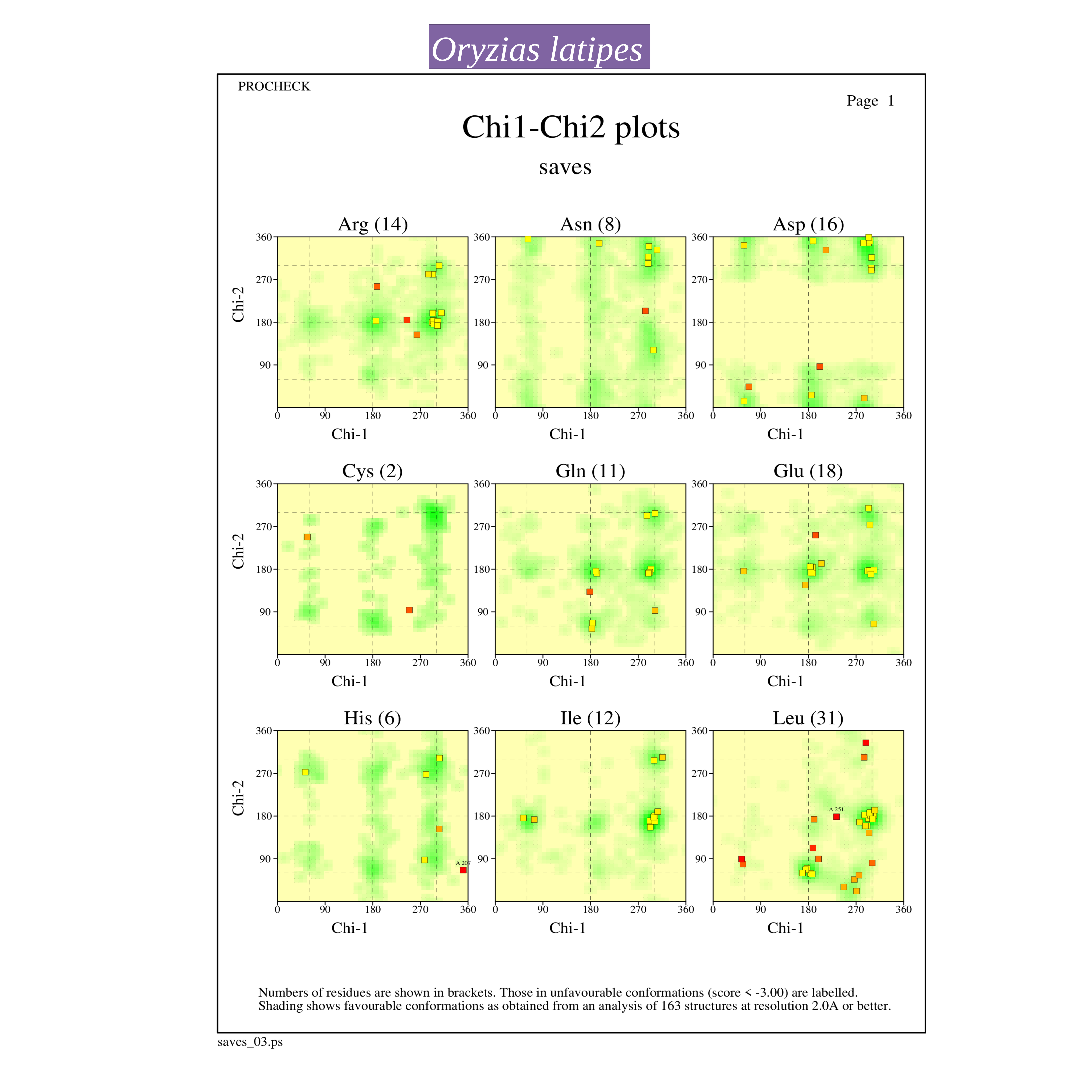

Oryzias latipes

## Slide 6
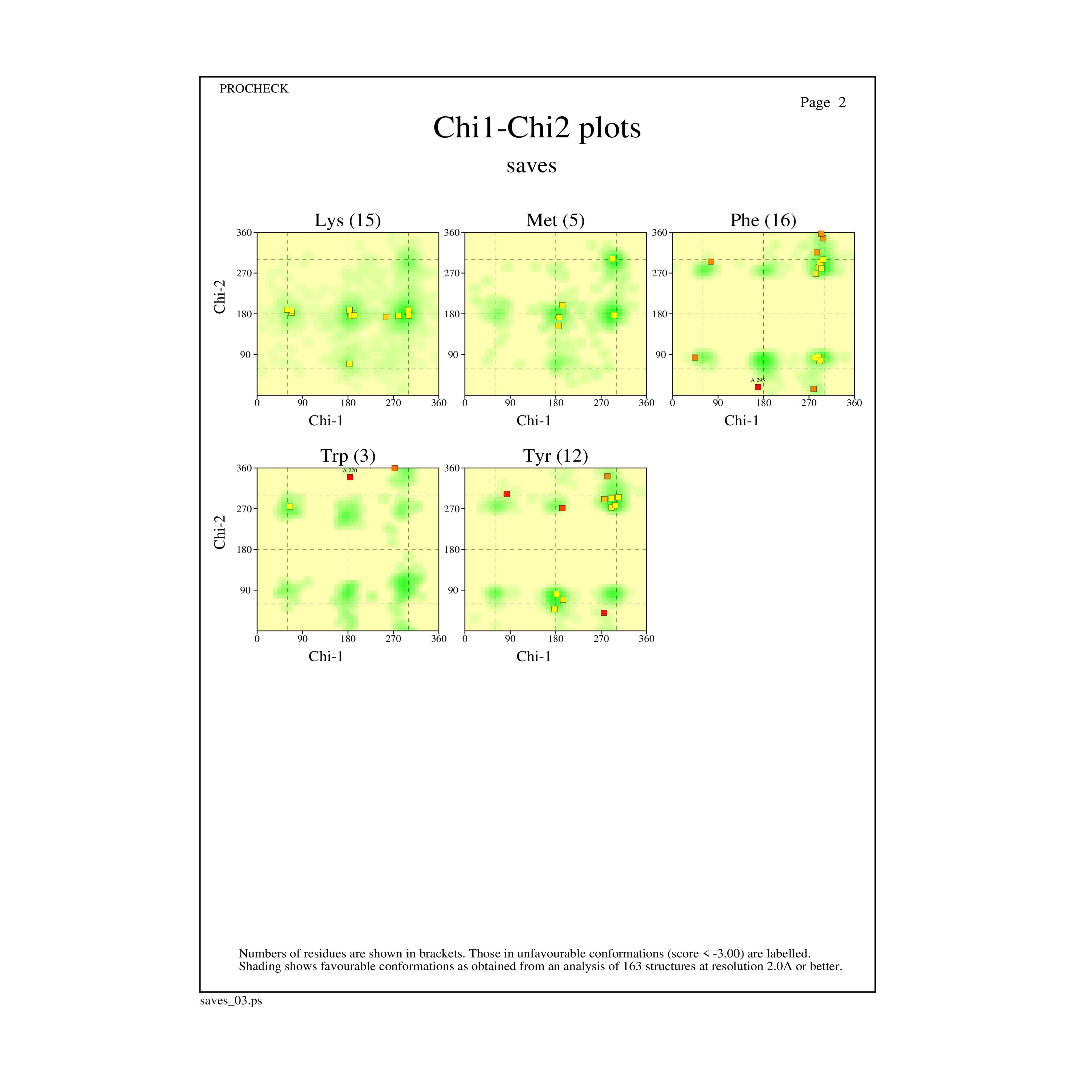

## Slide 7
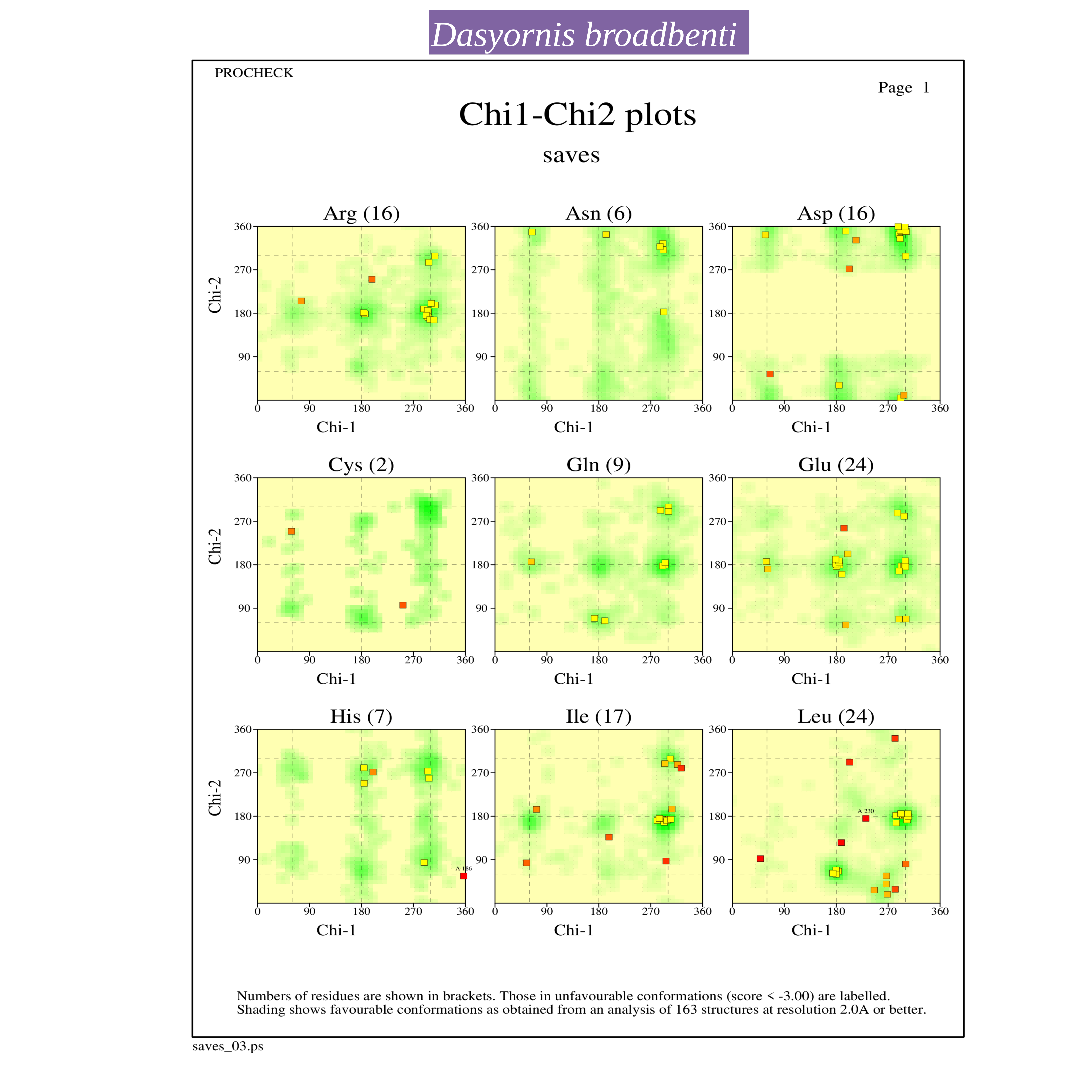

Dasyornis broadbenti

## Slide 8
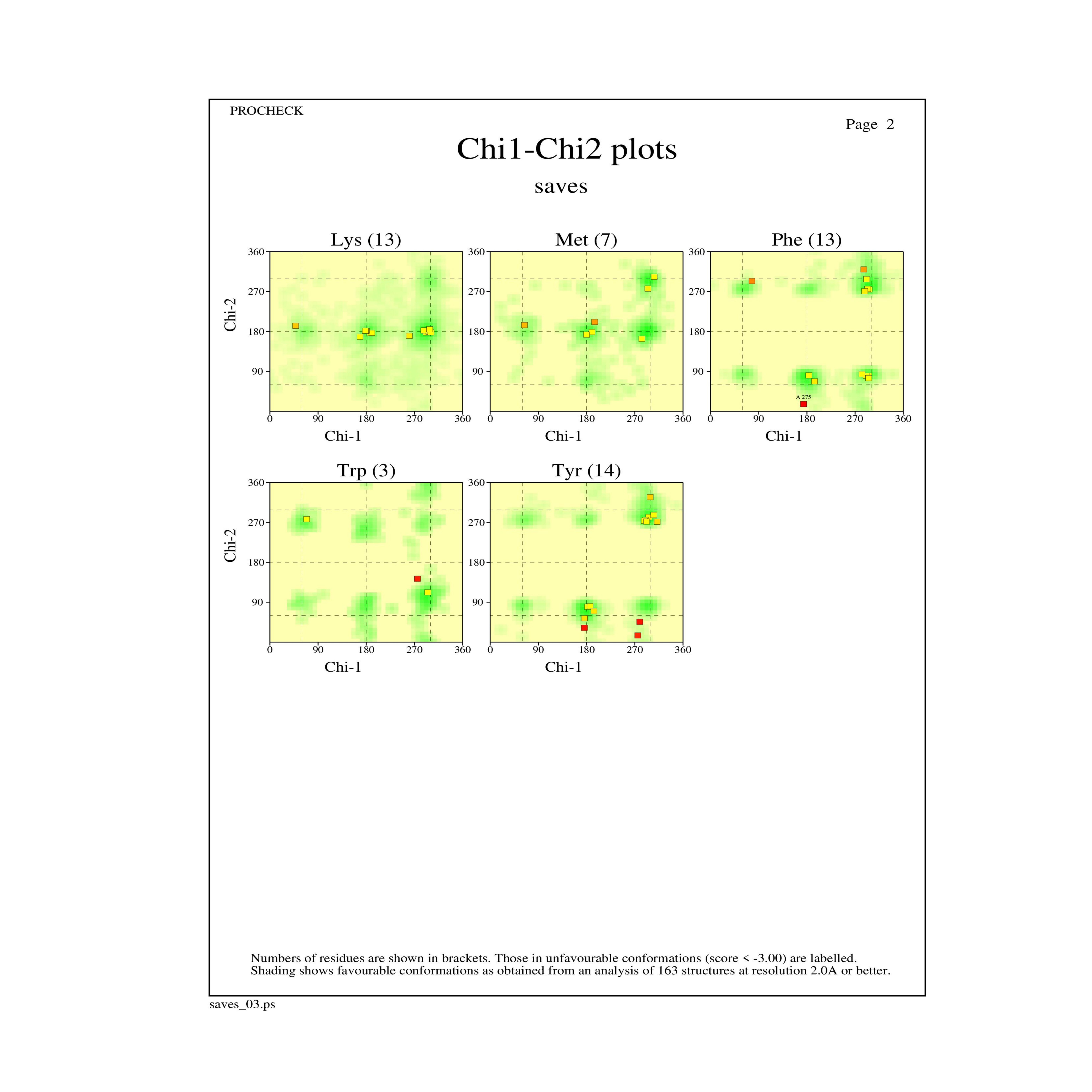

## Slide 9
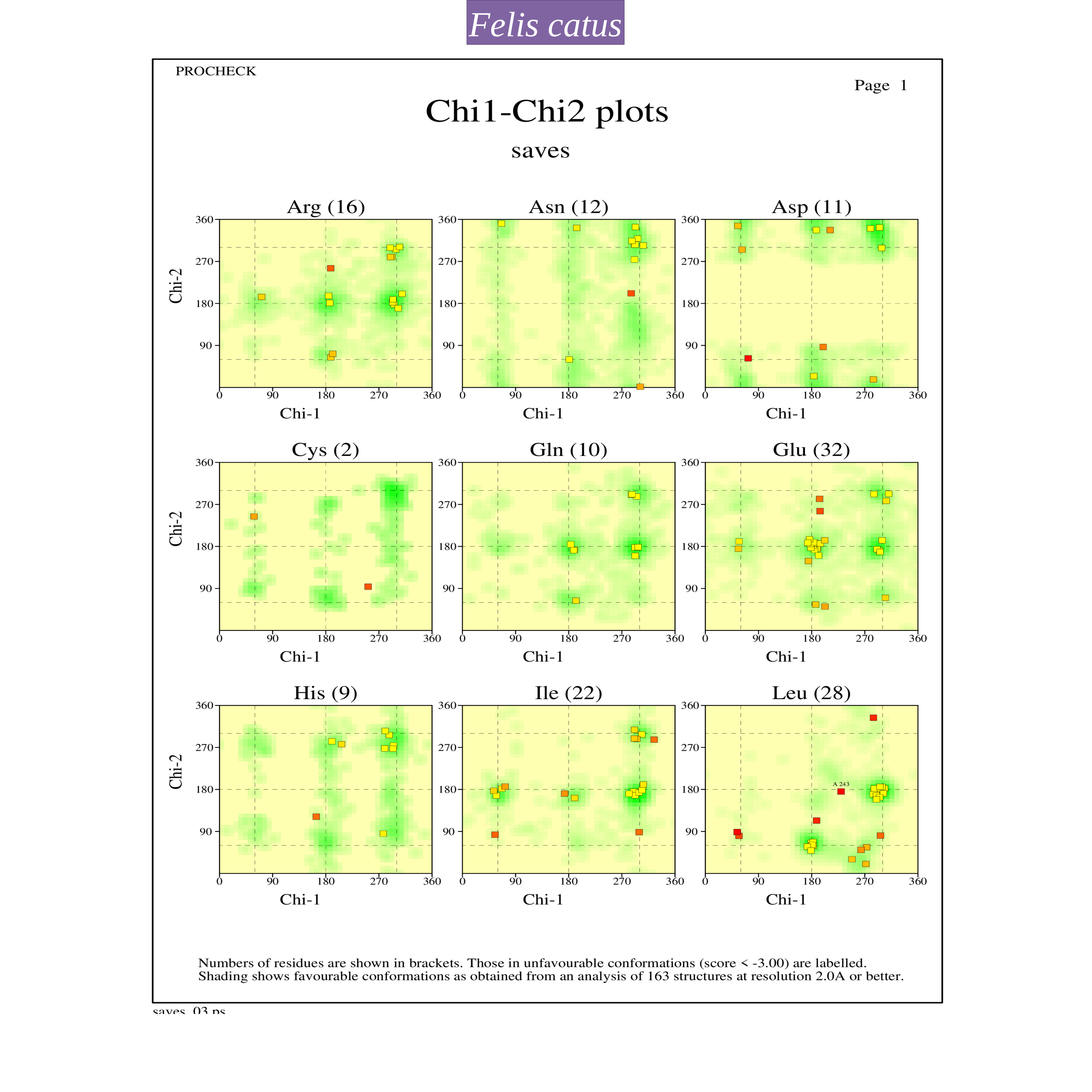

Felis catus

## Slide 10
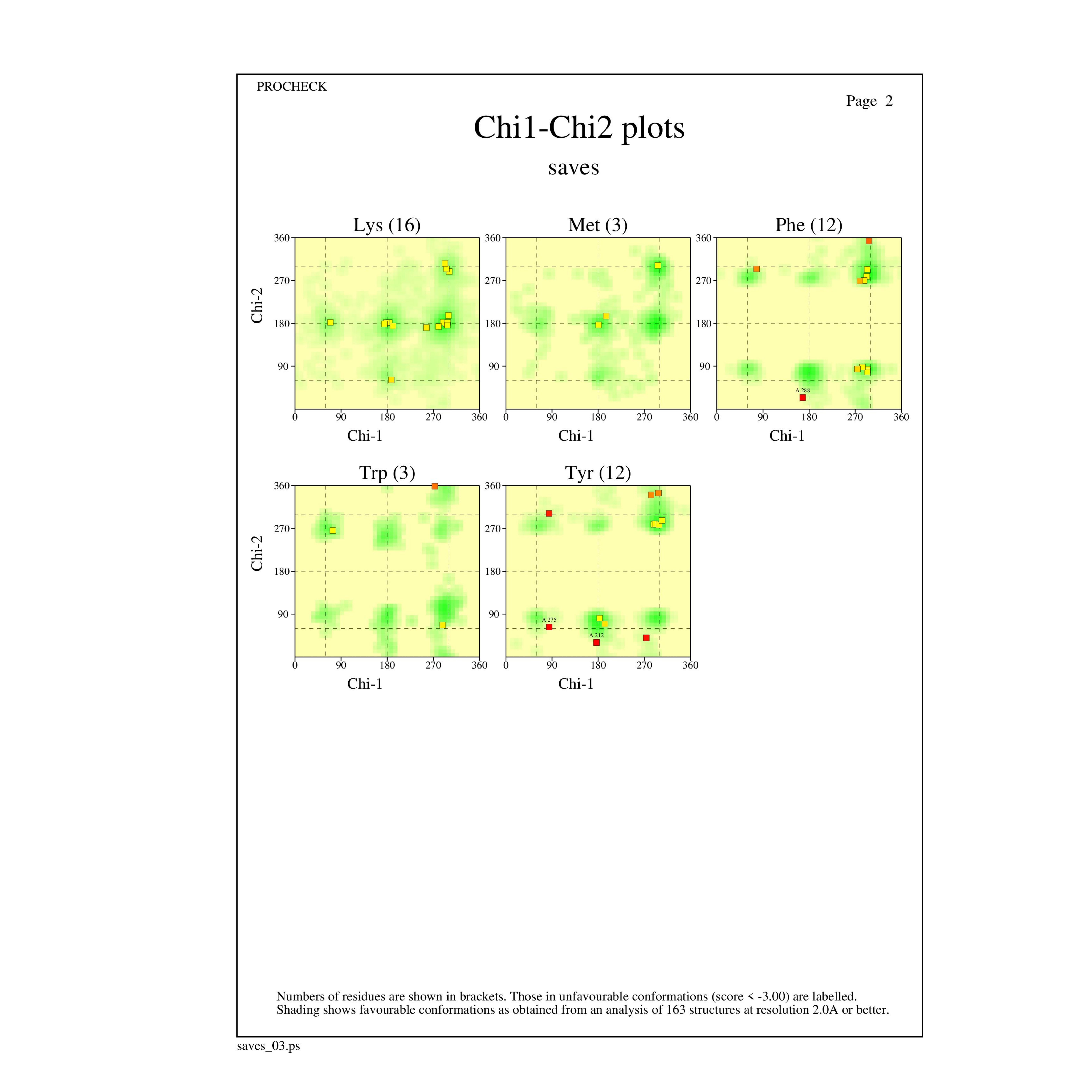

Supplement: Supplementary file 1 — Additional file 1. List of organisms with protein accession numbers. [file 43141_2023_522_MOESM1_ESM.pptx]
