# Supplementary figures and images for: In silico comparative structural and functional analysis of arsenite methyltransferase from bacteria, fungi, fishes, birds, and mammals
Source: J Genet Eng Biotechnol. 2023 May 19;21:64. doi: 10.1186/s43141-023-00522-9 (PMC10199152; doi:10.1186/s43141-023-00522-9)

## Slide 1
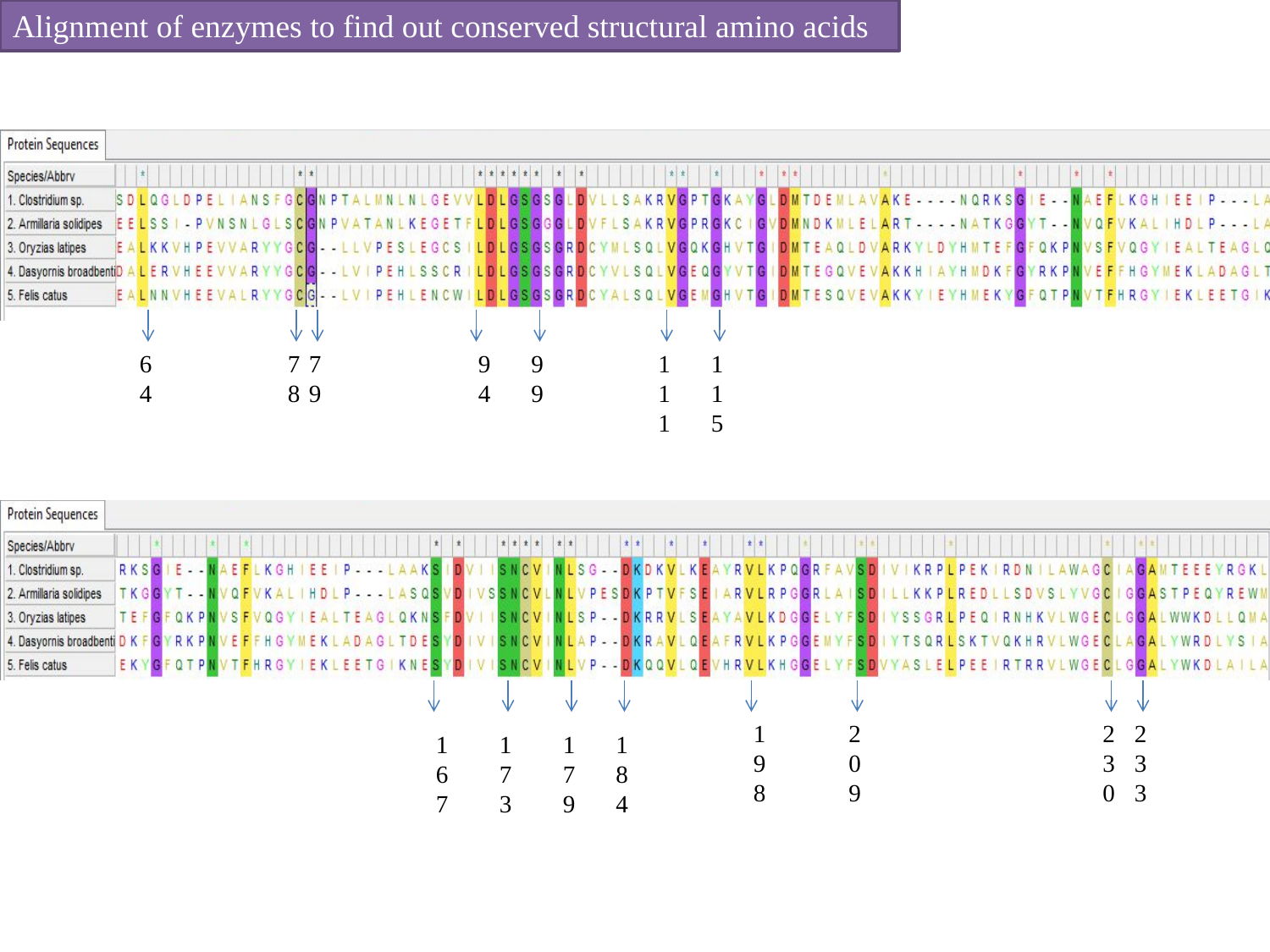

Alignment of enzymes to find out conserved structural amino acids
64
78
79
94
99
111
115
198
209
230
233
167
173
179
184

Supplement: Supplementary file 3 — Additional file 3. Phylogenetic trees of different organisms. [file 43141_2023_522_MOESM3_ESM.pptx]
