# Supplementary material for: In silico comparative structural and functional analysis of arsenite methyltransferase from bacteria, fungi, fishes, birds, and mammals
Source: J Genet Eng Biotechnol. 2023 May 19;21:64. doi: 10.1186/s43141-023-00522-9 (PMC10199152; doi:10.1186/s43141-023-00522-9)
Supplement: Supplementary file 7 — Additional file 7. Ramachandran plots of individual amino acids of selected enzymes. [file 43141_2023_522_MOESM7_ESM.pptx]

## Slide 1
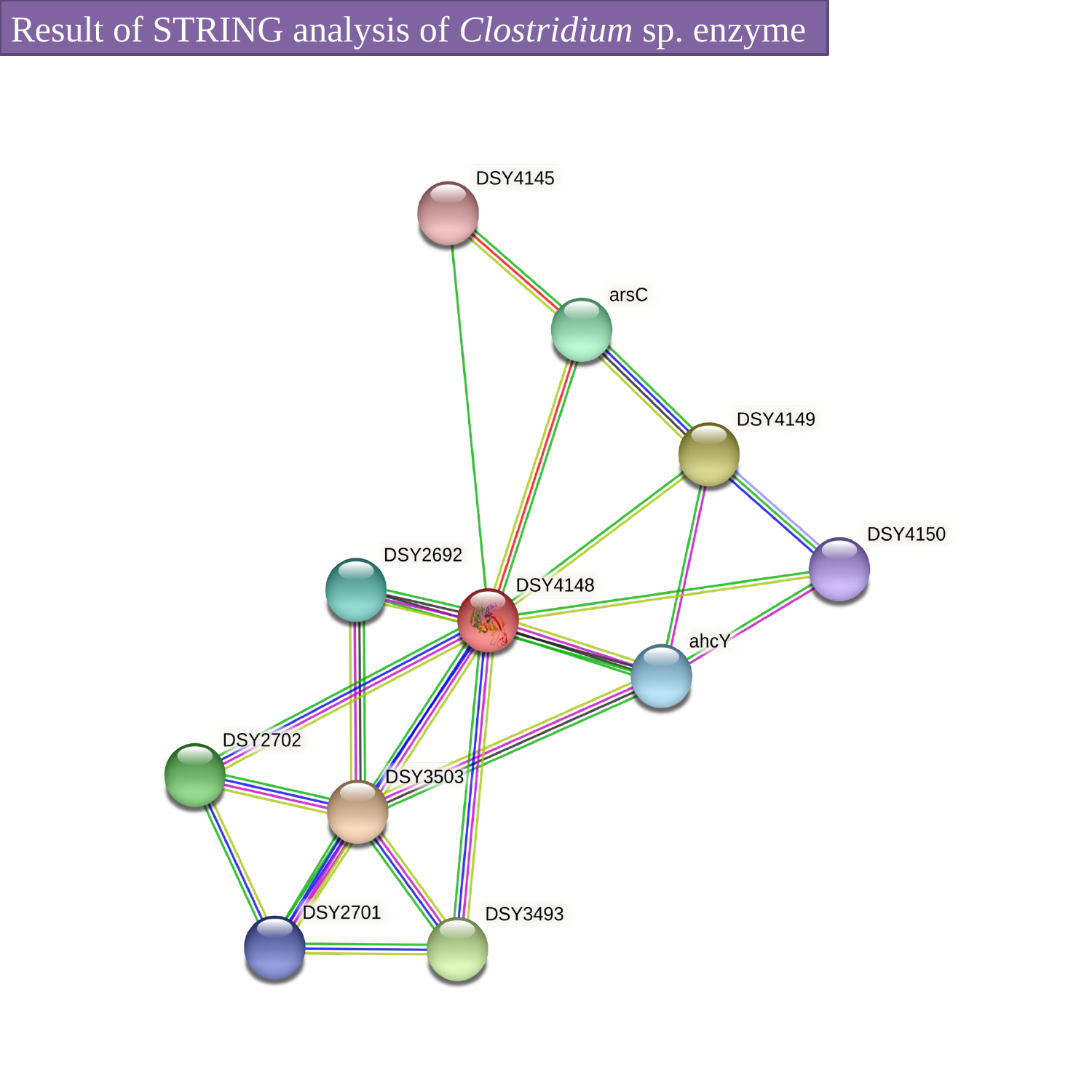

Result of STRING analysis of Clostridium sp. enzyme

## Slide 2
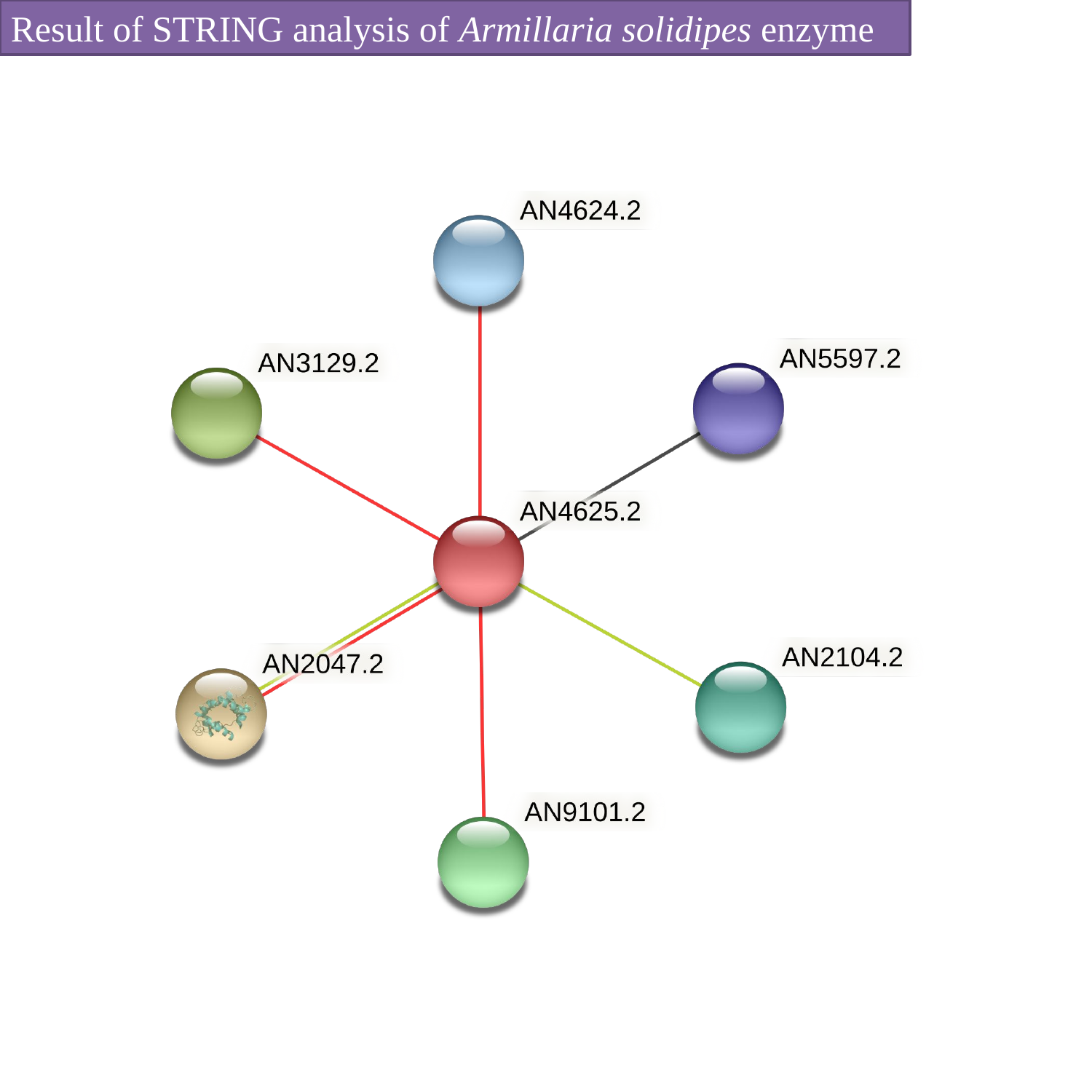

Result of STRING analysis of Armillaria solidipes enzyme

## Slide 3
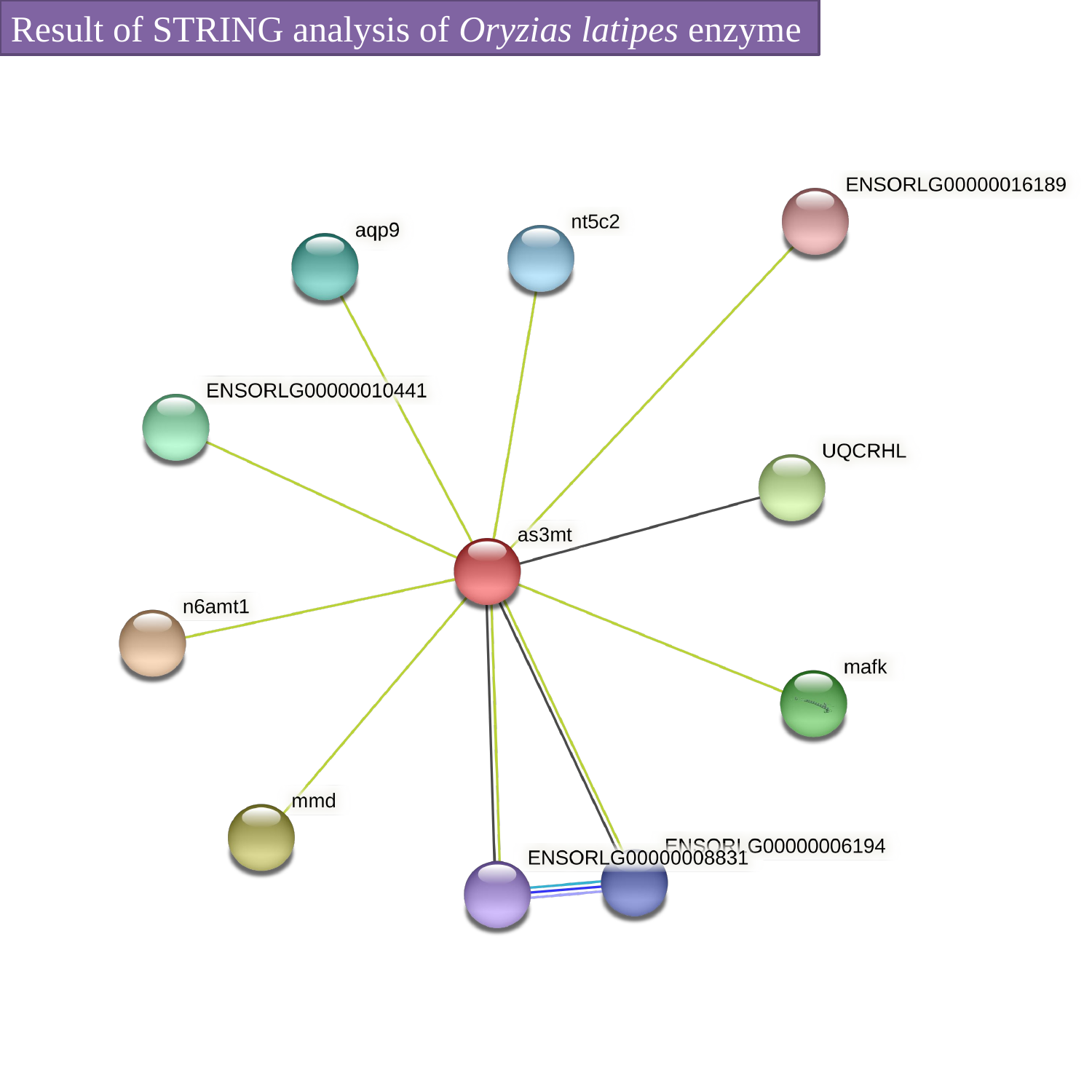

Result of STRING analysis of Oryzias latipes enzyme

## Slide 4
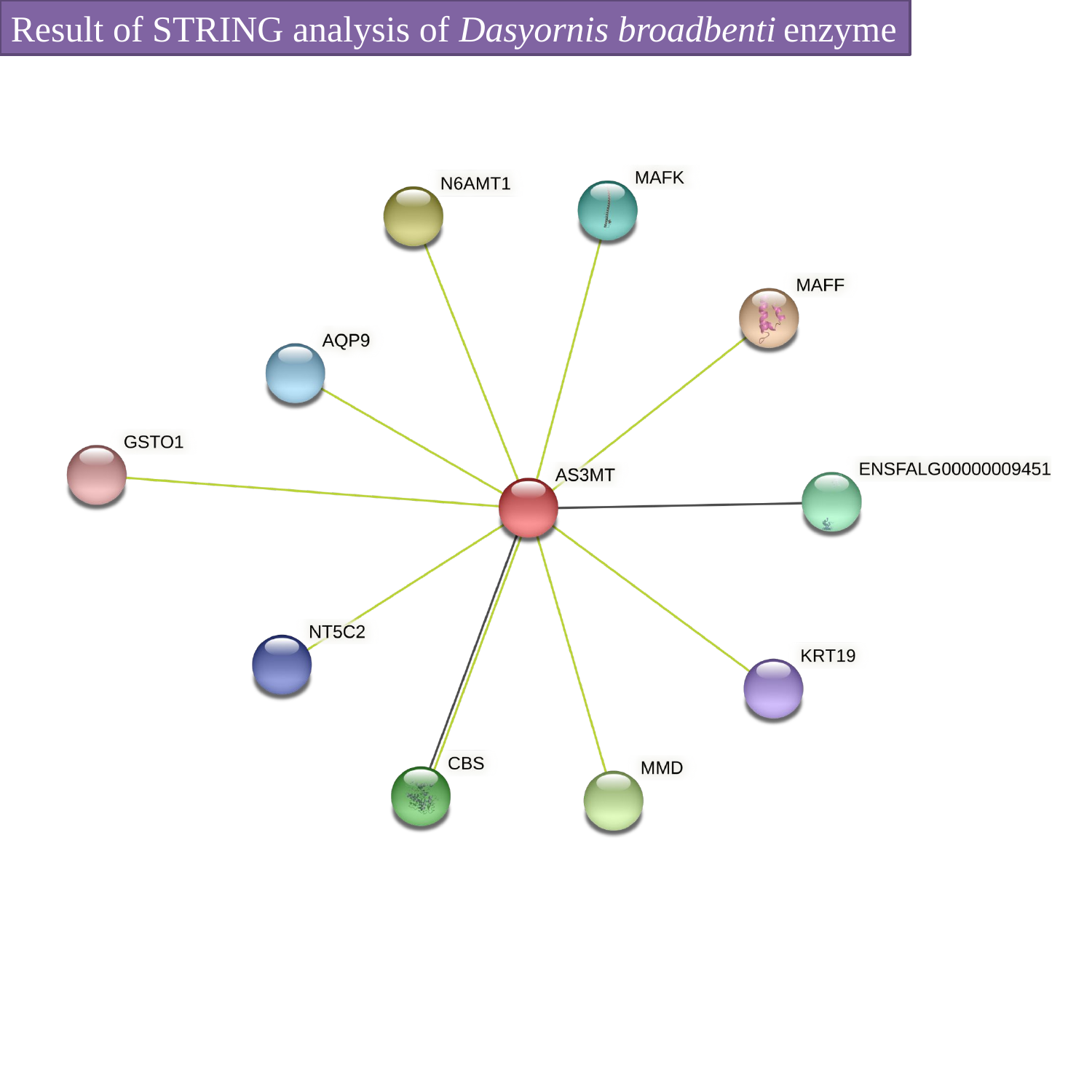

Result of STRING analysis of Dasyornis broadbenti enzyme

## Slide 5
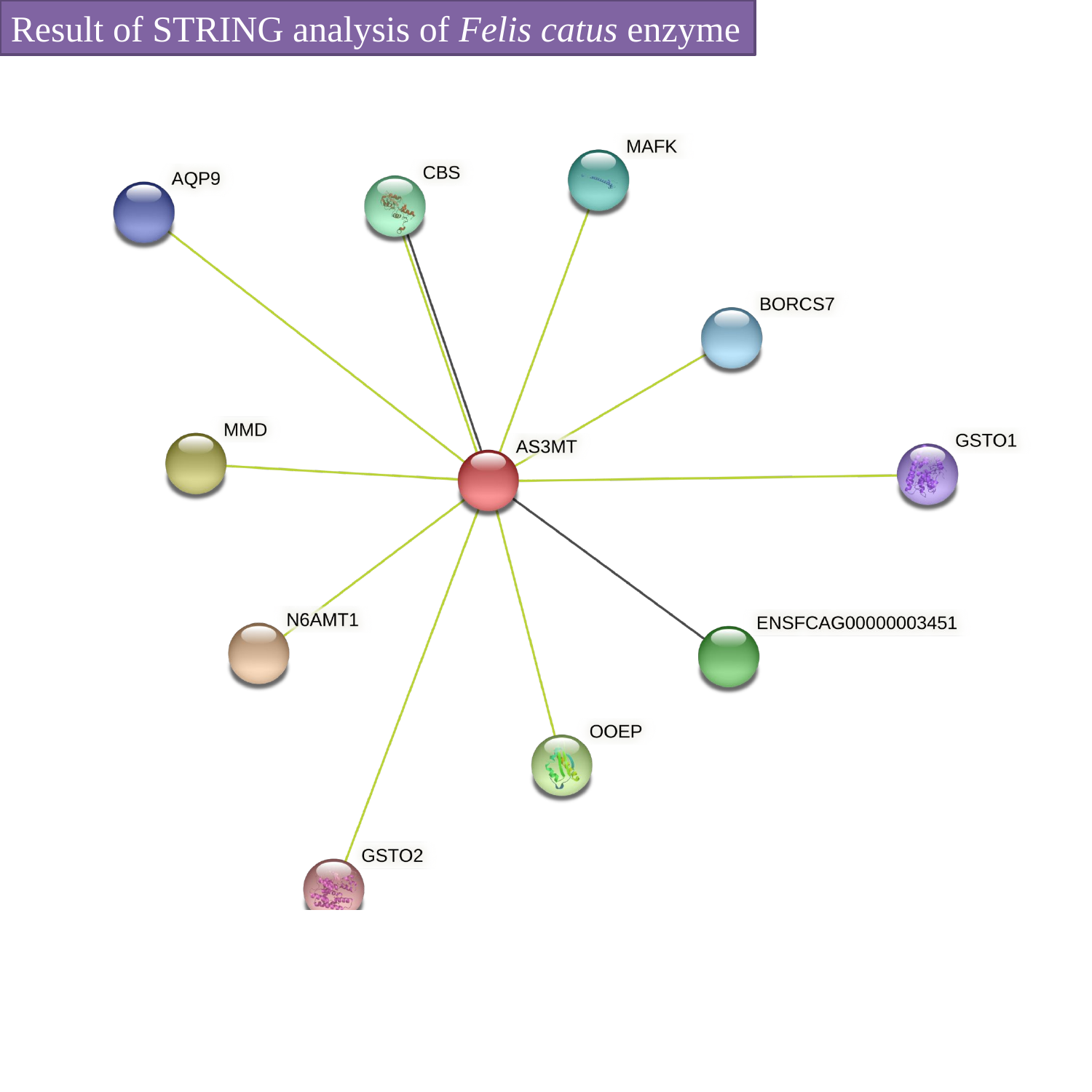

Result of STRING analysis of Felis catus enzyme
