# Supplementary material for: In silico comparative structural and functional analysis of arsenite methyltransferase from bacteria, fungi, fishes, birds, and mammals
Source: J Genet Eng Biotechnol. 2023 May 19;21:64. doi: 10.1186/s43141-023-00522-9 (PMC10199152; doi:10.1186/s43141-023-00522-9)
Supplement: Supplementary file 11 — Additional file 11. Identification of probable motifs. [file 43141_2023_522_MOESM11_ESM.docx]

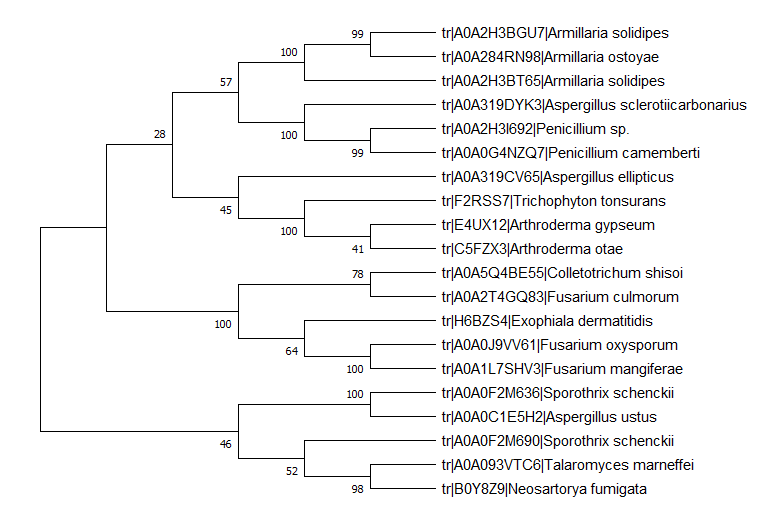

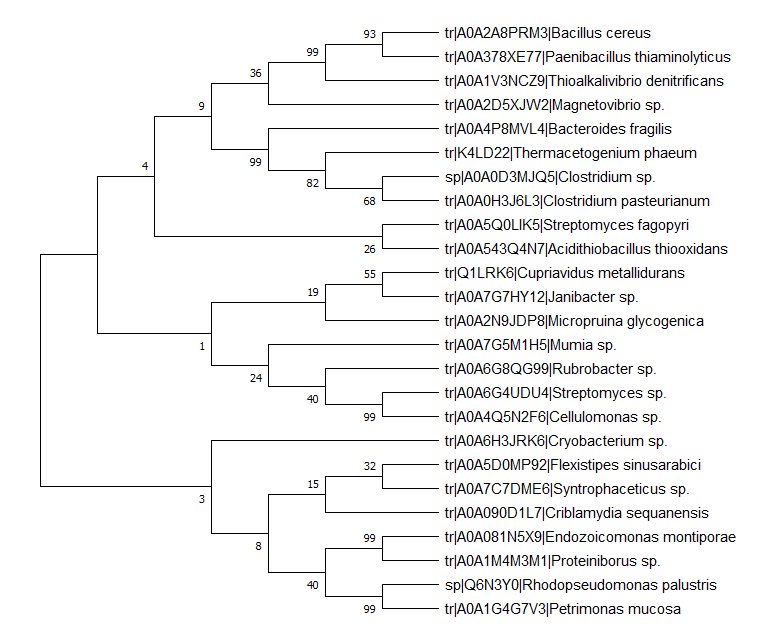
 **Fig. S3.1**: Phylogenetic tree of arsenite methyltransferase of bacteria

**Fig. S3.2**: Phylogenetic tree of arsenite methyltransferase of fungi


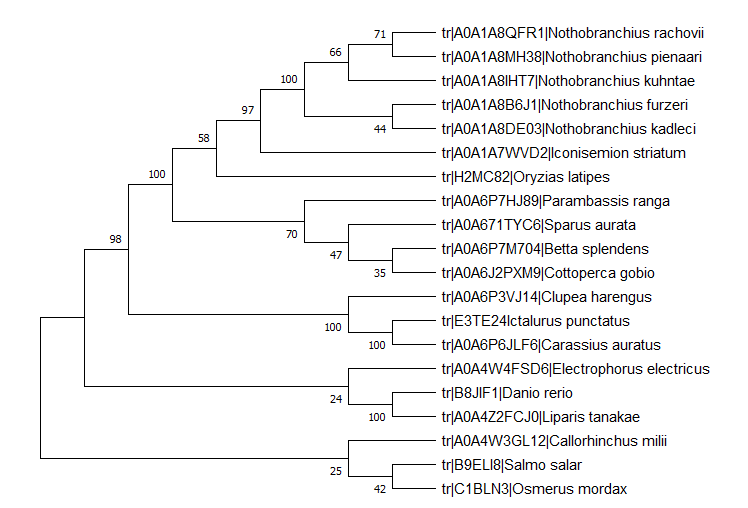


**Fig. S3.3**: Phylogenetic tree of arsenite methyltransferase of fishes

**
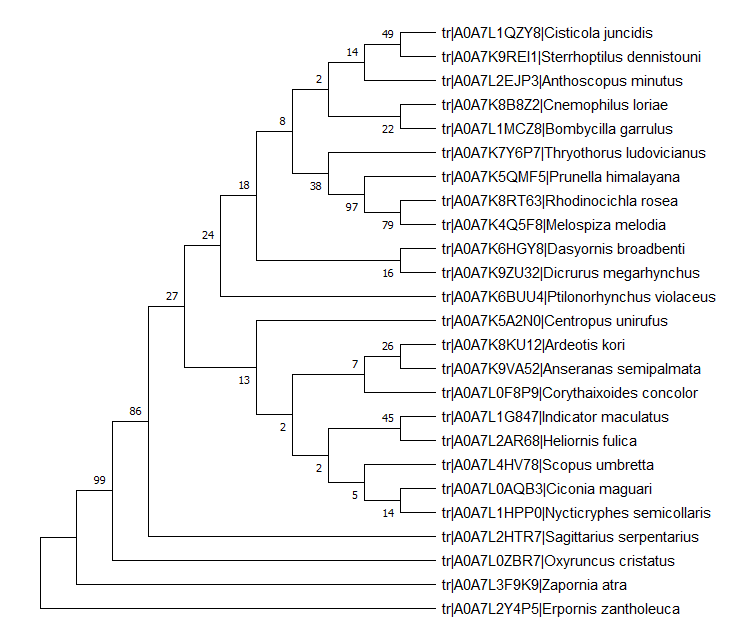
**

**Fig. S3.4**: Phylogenetic tree of arsenite methyltransferase of birds

**Fig. S3.5**: Phylogenetic tree of arsenite methyltransferase of
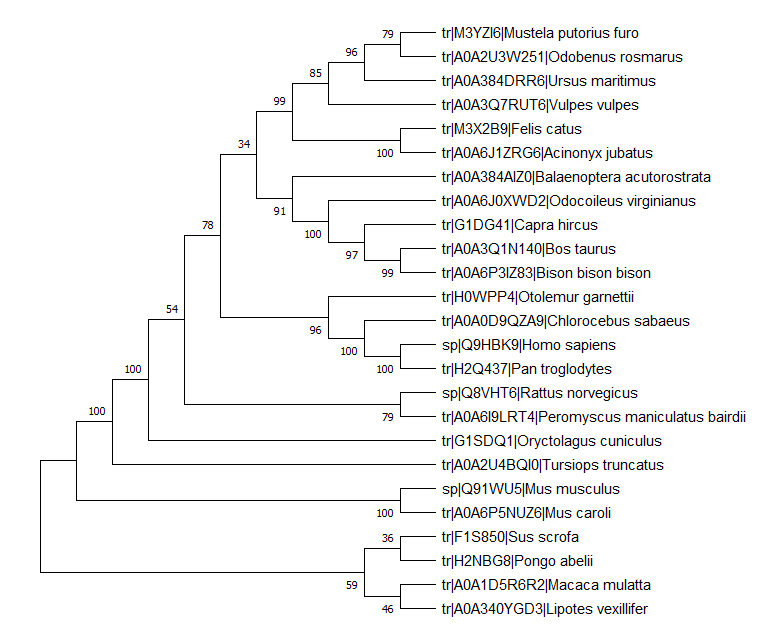
mammals
