# Supplementary material for: In silico comparative structural and functional analysis of arsenite methyltransferase from bacteria, fungi, fishes, birds, and mammals
Source: J Genet Eng Biotechnol. 2023 May 19;21:64. doi: 10.1186/s43141-023-00522-9 (PMC10199152; doi:10.1186/s43141-023-00522-9)
Supplement: Supplementary file 12 — Additional file 12. Results of STRING analysis. [file 43141_2023_522_MOESM12_ESM.pptx]

## Slide 1
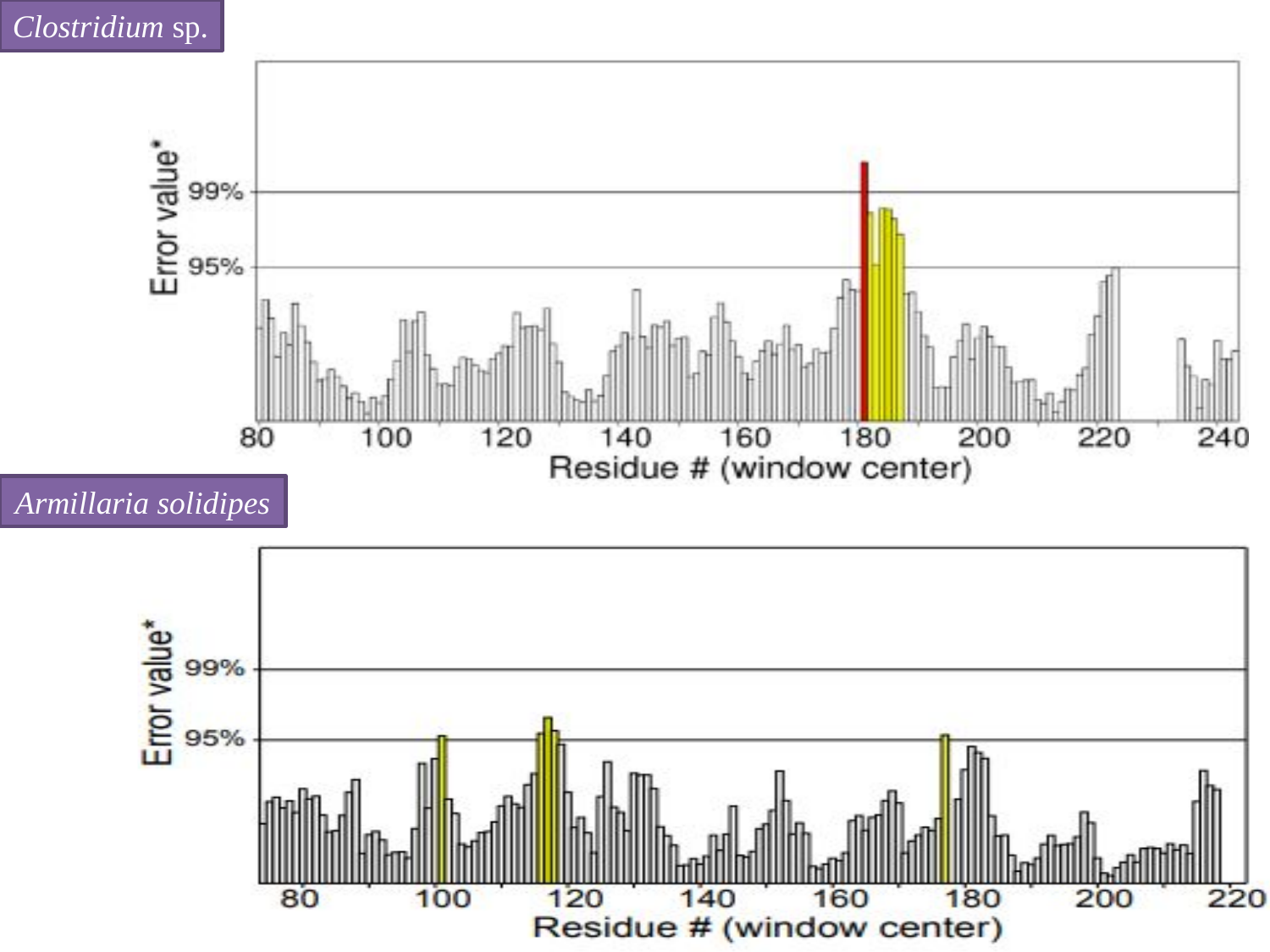

Clostridium sp.
Armillaria solidipes

## Slide 2
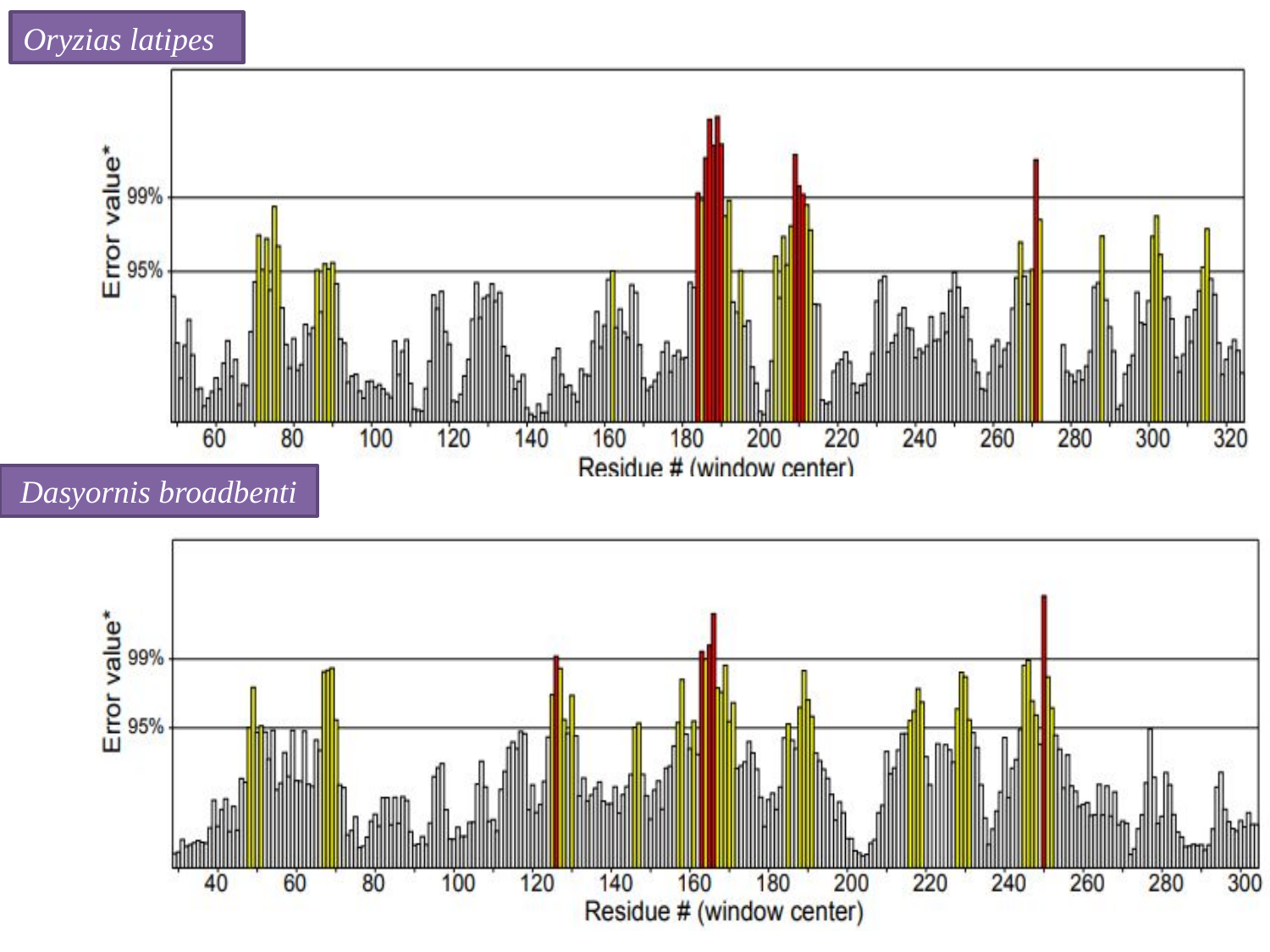

Oryzias latipes
Dasyornis broadbenti

## Slide 3
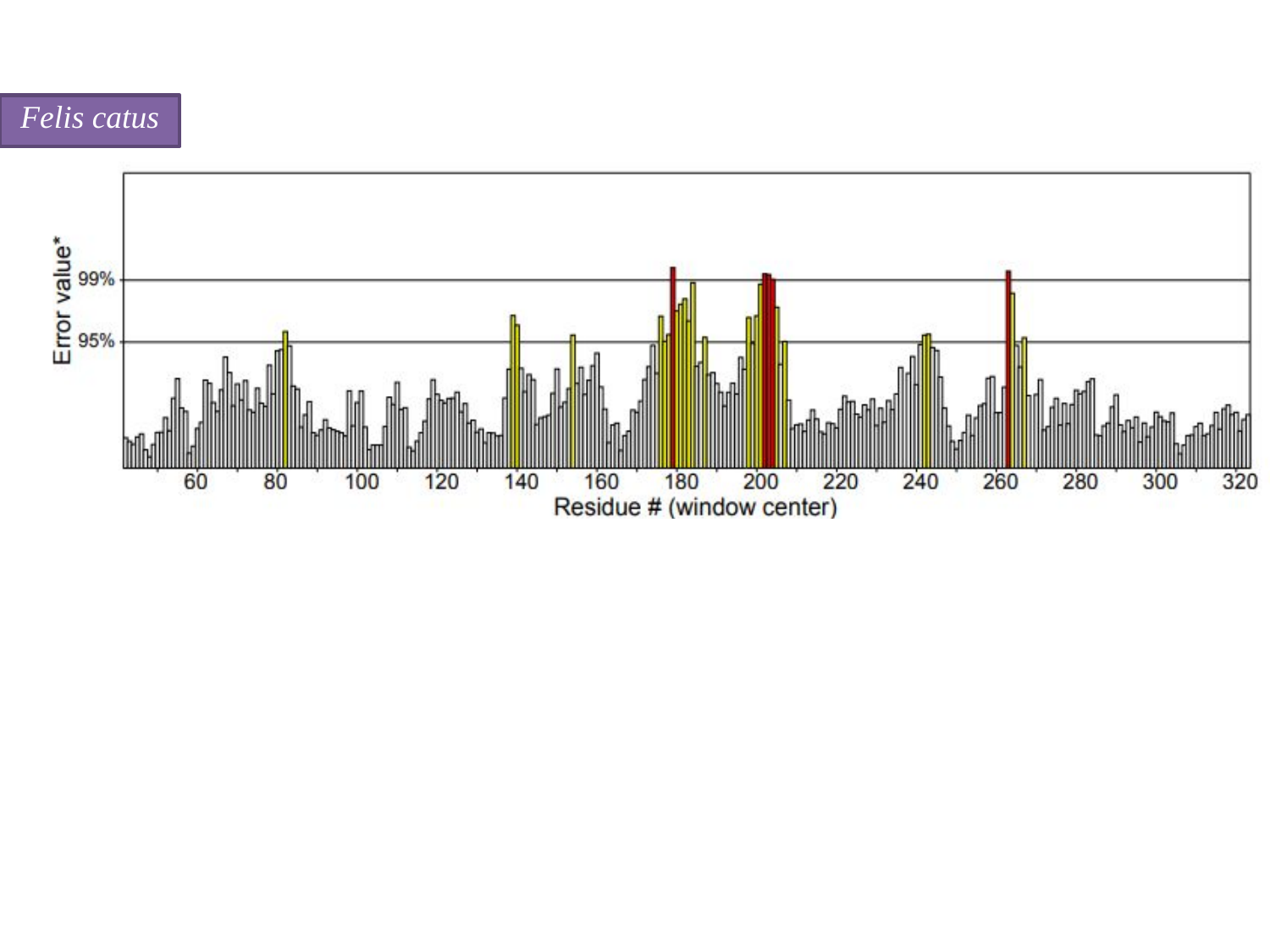

Felis catus
